# Countering Authoritarian Behavior in Democracies

**DOI:** 10.1007/s11109-024-09971-5

**Published:** 2024-09-13

**Authors:** Sara B. Hobolt, Moritz Osnabrügge

**Affiliations:** 1 London School of Economics and Political Science, London, UK; 2 Durham University, Durham, UK

**Keywords:** Counteractions, Authoritarian behavior, Candidates, Conjoint experiment, Democratic backsliding

## Abstract

Democratic stability hinges on voters’ commitment to democratic norms, yet there are many examples of voters’ willingness to tolerate politicians who violate such principles. This article examines whether critical responses by other politicians can effectively counter the appeal of political candidates who have engaged in authoritarian behavior. We argue that costly action by fellow partisan politicians can reduce the electoral popularity of authoritarian politicians. We test this in a preregistered conjoint experiment embedded in a nationally representative survey in the United Kingdom. The results show that voters are less likely to choose politicians displaying authoritarian behavior, when they are criticized by other legislators, and that such counteractions are particularly effective when they are costly. These findings have important implications, as they show that politicians, especially co-partisans, can play an important role in reducing the appeal of authoritarian politicians.

**Supplementary Information:**

The online version contains supplementary material available at 10.1007/s11109-024-09971-5.

## Introduction

In recent years, populist and authoritarian politicians have gained momentum in many Western democracies. Studies have shown that voters may tolerate attacks on democratic values in return for partisan victories, policy benefits, and the election of competent candidates (Carey et al., 2022; Frederiksen, 2022b; Graham & Svolik, 2020; Gratton & Lee, 2024; Krishnarajan, 2023). The tolerance, and even appeal, of authoritarian politicians is worrying in developed democracies, as it may lead to the gradual erosion of democratic norms (Dahl, 1961). While threats to established liberal democracies are increasingly well-documented (Burgoon et al., 2019; De Vries & Hobolt, 2020; Grossman et al., 2022; Kelemen, 2017; Levitsky & Ziblatt, 2018; Mounk, 2018; Norris & Inglehart, 2019; Przeworski, 2019), we know less about the effectiveness of actions to counter authoritarian tendencies.

This article examines whether the responses of fellow politicians can effectively reduce the electoral appeal of politicians displaying authoritarian behavior, and what type of “counteractions” are most effective. Following Levitsky and Ziblatt (2018, pp. 23–24), authoritarian behavior occurs when politicians (i) reject or only weakly commit to democratic rules, (ii) deny legitimacy of political opponents, (iii) tolerate or encourage violence, and/or (iv) are ready to limit civil liberties of opponents. Such behavior is found both on the political extremes as well as within the ranks of mainstream parties, including the recent prominent examples of Donald Trump in the United States, Victor Orbán in Hungary, Benjamin Netanyahu in Israel, and Boris Johnson in the United Kingdom (Kelemen, 2017; Levitsky & Ziblatt, 2018; Norris & Inglehart, 2019).

Given that voters are likely to discount most criticism by other politicians as simply part of the cut and thrust of political debate, we argue that the effectiveness of counteractions depends on how credible they appear to voters (Berinsky, 2017; Druckman, 2001; Hovland et al., 1953; Lupia & McCubbins, 1998). An important aspect of perceived credibility is observable costly effort (Cho & Kreps, 1987; Cukierman & Tommasi, 1998; Spence, 1973; Wagner et al., 2020). When it comes to counteractions, there are two important components of observable costly action. The first is the actor initiating the counteraction and the second is the counteraction itself: it is more costly to criticize a politician from within your own ranks, i.e. a co-partisan, than criticize someone from an out-party. This means that counteractions by fellow partisans are likely to be more effective in persuading voters. The second is the severity of the counteraction itself. The costlier the action is to the messenger, the more credible and thus persuasive it will be in reducing the appeal of authoritarian politicians.

To analyze the effectiveness of counteractions, we design a conjoint experiment, conducted on a nationally representative sample in one of the world’s oldest democracies, the United Kingdom. As a long-standing democracy, which has nonetheless experienced instances of politicians seeking to jeopardize core liberal democratic norms,^1^ the United Kingdom is a good case for examining the effectiveness of counteractions. Our preregistered conjoint experiment allows us to uncover the relative influence of different factors in candidate choice in elections (Bansak et al., 2023; Hainmueller et al., 2014; Leeper et al., 2020). We present respondents with candidates who have been embroiled in a range of different controversies, where some pertain to authoritarian views or behaviors and others relate to common misdemeanors among Members of Parliament (MPs) such as misuse of public funds, extramarital affairs and indifference to constituents. Importantly, we also randomize the responses (counteractions) by other MPs to these controversies, ranging from support for the candidate to calling for their resignation. The experimental design thus allows us to estimate the causal effect of the candidates’ behavior and the effectiveness of different counteractions—varying both in terms of the actors (in-party or out-party) and the severity of the criticism—in shaping vote intention and candidate approval.

Our findings reveal that voters are not more likely to punish politicians engaged in authoritarian behavior compared to those engaged in any other misdemeanors. However, we do find that such behavior is less likely to be tolerated if criticized by other politicians, especially if these counteractions are costly. Hence, while our results show that voters may be willing to tolerate authoritarian behavior compared to misdemeanors, the evidence also suggests that counteractions by mainstream politicians, especially co-partisans, can be effective in combating authoritarian tendencies within parties.

Our contribution is thus two-fold. First, we contribute to the literature on democratic backsliding by developing a theoretical argument and empirical test of the effectiveness of counteractions to authoritarian behavior. Recent research identifies various strategies for countering democratic backsliding (Bellamy & Kröger, 2021; Devine, 2023; Kelemen, 2017; Levitsky & Ziblatt, 2018; Lührmann, 2021; Somer et al., 2021), but few studies analyze the effectiveness of different strategies on voters’ willingness to tolerate politicians with authoritarian tendencies.^2^ We thus expand upon burgeoning research on democratic backsliding by studying the effectiveness of counteractions by mainstream parties in decreasing support for authoritarian politicians.

Second, our study increases our understanding of the factors influencing candidate choice in elections. Previous empirical studies on candidate choice emphasize how features of candidates, such as party affiliation, gender, race and policy positions, influence vote choice (e.g., Bansak et al., 2021; de la Cuesta et al., 2022; Eggers et al., 2018; Hainmueller et al., 2014; Robinson, 2023; Teele et al., 2018). In this article, we complement this vast literature on candidate choice, as we study how reactions of other politicians shape vote choice and thereby illustrate a novel application of conjoint experiments.

## The Impact of Counteractions on Candidate Choice

The threat to democratic institutions often comes from within, as populist and authoritarian politicians seek to subvert democratic institutions and norms. In established democracies, politicians with authoritarian tendencies rarely speak out against democracy per se. Instead, they generally present themselves as democrats while undermining core democratic norms by rejecting the “rules of the gam”, denying the legitimacy of opponents, tolerating or even encouraging violence, and curtailing core democratic institutions, such as the media and courts (Levitsky & Ziblatt, 2018; Linz, 2000). Given such threats, the resilience of democracy relies on its core norms being shared and defended by the broader society (Dahl, 1989; Weingast, 1997). In particular, it depends on elections as a selection mechanism, where voters reject politicians who openly seek to subvert such institutions and norms (Besley, 2006).

On the one hand, it may appear that elections alone provide an effective mechanism against any politicians with authoritarian positions, given the high and stable levels of support for democracy in the West. Studies consistently show that the vast majority of citizens prefer democracy over any other form of political system (Inglehart & Norris, 2003; Klingemann, 2014) and that a majority in the West believe that civil liberties and freedoms are key parts of democracy (Dalton et al., 2007). In longstanding democracies, most citizens learn about democratic norms and processes from a young age, making departures from these norms seem intuitively wrong (Becher & Brouard, 2022; Bor et al., 2021). Wuttke et al. (2022) reveal large, and stable, support for democracy in Western European countries, including in the United Kingdom, and Donovan (2019) demonstrates that even among voters of right-wing extremist parties over 75% of voters agree that democracy is good.

On the other hand, while citizens on the whole prefer democracy over other regime types when asked in surveys, recent research also suggests that the public in consolidated democracies fail to punish politicians who display authoritarian behavior (Chong, 1993; Donovan, 2019; Frederiksen, 2022c; Graham & Svolik, 2020; Gratton & Lee, 2024; Frederiksen, 2022a; Krishnarajan, 2023). For example, Graham and Svolik (2020) argue that Americans are willing to support politicians with whom they share policy preferences and partisanship, even when they take positions that violate core democratic principles. Their study demonstrates that American voters are more likely to reject hypothetical candidates who eschew their preferred policies than those who would violate norms of electoral fairness or checks and balances. In another experimental study, Carey et al. (2022) show that American voters will generally punish democratic transgressions among candidates that would undermine institutions of accountability. Yet, they also show that voters are more divided on other democratic norms, which suggests they may accept certain transgressions. In a comparative study of the United States, the United Kingdom, the Czech Republic, Mexico, and South Korea, Frederiksen (2022b) presents survey-experimental evidence to show that in all contexts support for undemocratic political leaders increases with their competence. On the whole, he concludes that “voters prefer undemocratic, competent candidates to democratically compliant, incompetent candidates” (Frederiksen, 2022b, p. 1147). Krishnarajan (2023) argues that citizens rationalize their perception of undemocratic behavior based on their policy preferences. This study presents evidence on democratic rationalization from the United States and 22 democracies.

This recent evidence thus suggests that some voters may be willing to tolerate, and even vote for, candidates engaged in authoritarian behavior, defined in this article as rejecting democratic rules, denying the legitimacy of political opponents, tolerating violence, and/or limiting civil liberties of opponents (Levitsky & Ziblatt, 2018; Przeworski, 2019; Zakaria, 1997). This raises the question of whether the responses to such behavior by other politicians can effectively reduce the appeal of authoritarian candidates.

A number of recent important studies have advanced our understanding of the type of counteractions available. Levitsky and Ziblatt (2018, pp. 24–26) argue that democratic politicians can isolate authoritarians by excluding them from party ballots, removing them from their grass roots, and avoiding alliances and collaboration. Lührmann (2021) identifies four main strategies for addressing authoritarian politicians: inclusion, counter-mobilization, ignoring, and exclusion (see also Somer et al., 2021). Furthermore, a number of recent articles put forward proposals for how to counter democratic backsliding in the European Union (Bellamy & Kröger, 2021; Kelemen, 2017).

While these studies advance our understanding of how politicians can seek to counter democratic backsliding, they do not test the effectiveness of different types of strategies on voters’ willingness to tolerate authoritarian politicians. Wuttke and Foos (2024) is a rare example of a study using a field experiment to show that politicians can increase public support for democracy by making arguments. Another recent experimental study by Voelkel et al. (2023) identifies several interventions, including correcting misperceptions of outpartisans’ views, that can reduce support for undemocratic practices. Clayton and Willer (2023) use experimental evidence to demonstrate that messages from Republican politicians defending the legitimacy of the 2020 US presidential election increased faith in the election’s outcome among Republican voters. Our article builds on these studies, and the literature on persuasion and signaling, to systematically test the effectiveness of different types of elite counteractions. In contrast to these earlier studies, we focus on the effect of counteractions on candidate choice rather than the factors influencing democratic attitudes.

Specifically, we are interested in examining whether critical responses by other politicians—or *counteractions*—can effectively reduce the appeal of authoritarian behavior. Counteractions may take many forms, but fundamentally they concern other politicians publicly criticizing the authoritarian behavior of a politician. Building upon Levitsky and Ziblatt (2018), we focus on three main counteractions to authoritarian candidates in elections: criticism of the candidate, refusing to work with the candidate, and calling for the candidate to be expelled from the legislature.

Regardless of the specific nature, counteractions by other politicians thus serve to draw attention to negative aspects of a candidate’s behavior or views. In general, we would therefore expect that criticism of a candidate’s behavior by other politicians will reduce their electoral appeal, whereas candidates who have other politicians speaking out in their defense will be more popular with voters. We expect that the very act of one politician condemning the acts of another has the potential to reduce the electoral appeal of the candidate who is being criticized. Primarily, this is because the counteraction sends a signal about the (poor) quality about the politician (Besley, 2006). When the criticism alerts attention to the fact that candidate’s behavior is a threat to democracy, it may also further serve to highlight to voters that candidate’s behavior is outside of democratic norms and encourage them to sanction the candidate accordingly (Levitsky & Ziblatt, 2018). Moreover, it is also plausible that the criticism can reduce the appeal of candidate as it sends a signal about their standing within the party and the parliament, which makes them seen as less effective legislators. Again, this is a signal about the lack of quality of the candidate that reduces their electoral appeal. This leads us to our first hypothesis.^3^

### H1:

Voters are less likely to prefer a candidate who has been isolated or whose views or actions have been criticized by other politicians (“counteraction”) than candidates who have not been criticized.

While we expect counteractions to reduce the appeal of those candidates who are the target of the criticism, not all criticism sends an equally strong signal to voters. Since the game of politics is one where politicians continuously criticize one another—especially their partisan opponents—many of such critiques are likely to be ignored or discounted by voters. Building on the literature on persuasion and signaling, we argue that the effectiveness of the counteraction depends on the degree of observable costly effort (Cho & Kreps, 1987; Cukierman & Tommasi, 1998; Gambetta, 2009; Lupia & McCubbins, 1998; Spence, 1973). If the criticism is without great cost to the messenger, e.g. an opposition politician criticizing a government party candidate, voters are more likely to discount the message. In contrast, if voters recognize that the counteraction is highly costly to the messenger, e.g. politicians threatening to expel one of their own party candidates, they will pay greater attention and find the counteraction more credible and persuasive. To assess the effectiveness of counteractions, we focus on two main dimensions along which cost can vary: the type of the actor engaging in a counteraction and the type of action.

Research has shown that the credibility of the messenger influences the effectiveness of the message (Berinsky, 2017; Chiang & Knight, 2011; Cukierman & Tommasi, 1998; Druckman, 2001; Hovland et al., 1953; Lupia & McCubbins, 1998). Politicians continuously criticize those candidates belonging to an opposing party, but criticisms are more credible if politicians make statements that “run contrary to their personal and political interests” (Berinsky, 2017, p. 242). Politicians countering a co-partisan may face personal and political costs, which can credibly signal to voters that the candidate is a threat to democracy. For example, these costs can be career costs as parliamentarians need the support of co-partisans to advance in their political career (Norton, 2013). This is likely more pronounced in parliamentary systems, where party discipline and cohesion is higher as legislators depend on the approval by party elites for re-election and for privileged positions within parliament and elsewhere (Carey, 2007; Proksch & Slapin, 2012). Also, countering a co-partisan may damage the party reputation or profile, which plays a crucial role in shaping voting decisions in the UK (e.g., Denver & Johns, 2022; Greene & Jennings, 2017). Moreover, in competitive partisan politics where politicians from opposite sides repeatedly take the opportunity to criticize each other, it is far more informative to a voter when a co-partisan politician criticizes their peer than when a politician is met with the usual attacks by “the other side”. We thus expect that counteractions from in-group partisans are more likely to reduce the appeal of the candidate facing the criticism.

### H2:

Voters are less likely to prefer a candidate countered by politicians from the same party than a candidate countered by politicians from another party.

Note that following this logic, the link between the partisan affiliation of the messenger and the credibility of the message not only applies to criticism (counteractions), but also to supportive and neutral actions. In other words, politicians from the same party are expected to be *less* credible in comparison to politicians from another party when they act *in support* of a candidate, since it is far less costly for co-partisans to support each other than for opposing politicians to do so. Therefore, the negative effect of co-partisan affiliation is expected to exist for counteractions, supportive, and neutral actions.

The second component of a costly signal is the type of action itself. Politicians can criticize the candidates’ action as damaging democracy or take stronger actions such as committing to not work with the candidate and calling for the candidate to be expelled from the party. These counteractions vary in terms of the degree to which they are “costly” for the messenger to send (Gambetta, 2009; Lupia & McCubbins, 1998; Wagner et al., 2020). A costly message can reveal to the voter information on threats related to the candidate. At one end of the spectrum is simply criticizing a candidate without taking any action. This is done constantly in politics, and is more likely to be discounted by voters. At the other end of the spectrum are actions that carry a greater potential cost to the counteracting politician, such as refusing to work with a candidate or calling for the candidate to be expelled. These actions are costly because of commitment costs. More specifically, voters may punish parliamentarians in the future if parliamentarians change their position or the message turns out to be exaggerated (e.g., Andreottola, 2021; Fearon, 1994). Also, these actions may involve career costs and lost policy-making opportunities, since as effective legislative work often requires (cross-party) collaboration, e.g. in committees (Betsy & Goldsmith, 2019; Norton, 2013).

### H3:

The more severe the counteraction, the greater the effect on voters’ likelihood of preferring a candidate.

## Methods

We test these arguments in a preregistered conjoint experiment. Conjoint analysis is particularly well-suited to examine the effect of counteractions in electoral politics for multiple reasons (Bansak et al., 2023). First, the experimental design captures the multidimensionality of electoral choices and other complex opinion formation processes (Frederiksen, 2022b; Hahm et al., 2019; Hainmueller et al., 2014). Second, the design allows estimating a meaningful quantity of interest, the Average Marginal Component Effect (AMCE). This quantity corresponds to the average probability change of choosing a politician given a change of attributes and the expected change in vote share. Lastly, the AMCE can be estimated using minimal assumptions and non-parametric methods (see also Hainmueller et al., 2014; Leeper et al., 2020).

In a conjoint candidate study, participants are shown a series of pairs of candidates that vary according to a set of features, with combinations of features randomly varied. Respondents then select which of the two candidates they prefer. Rather than asking people directly about each separate feature, their discrete choices reveal the acceptability of different features. Since conjoint profiles vary along multiple dimensions, conjoint experiments have been shown to reduce social desirability (Horiuchi et al., 2022). This is important in this context, since respondents are not asked to directly express their opinions regarding authoritarian behavior and counteractions, but rather they reveal their preferences through the candidate choice and rating.

We implemented our survey-embedded conjoint experiment using the reputable survey company Deltapoll, which recruited a representative sample of English voters.^4^ Table A1 in Appendix A compares our sample to the UK census and shows the distribution in the population and our sample are very similar.

Britain is a suitable case for a number of reasons. Firstly, it is an old and well-established democracy, where we would expect voters to be sensitive to the importance of liberal democratic norms. Secondly, there has been a debate on politicians transgressing liberal democratic norms in recent years in the UK, which makes the experiment more realistic to respondents. A high-profile example of this is the 2019 controversy over the prorogation, or suspension, of Parliament by the Conservative Prime Minister Boris Johnson, which was later ruled unlawful by the Supreme Court (Grillo & Prato, 2023). At the time, the Speaker of the House, John Bercow, called it an “constitutional outrage” that would serve to “undermine [Johnson’s] democratic credentials and indeed his commitment to parliamentary democracy” (Proctor, 2019). Appendix H contains examples of the other controversies featured in our experiment, illustrating the external validity of these scenarios in a British context. Finally, the British case is well-suited to experimentally study the factors shaping candidate choice since voters in the UK vote directly on a candidate in their constituency (Frederiksen, 2022a). The British party system is also highly adversarial with two dominant competing parties^5^—the Conservatives and Labour—thus allowing us to examine the differences between in-party and out-party counteractions.

We preregistered our experimental design and hypotheses at OSF.^6^ The survey was fielded in February 2021. Our sample includes 4,012 respondents. The survey introduces respondents to the scenario and then shows them two candidates competing for election to Parliament in a general election. These candidates differ along six dimensions: the controversy, the actor reacting to the controversy, the reaction (counteraction), gender, party, and experience. The order of the dimensions varies randomly.^7^ After receiving information on two candidates, respondents choose one candidate and rate both. Each respondent participates in five conjoint tasks, which are illustrated on different screens. Thus, our dataset includes 40,120 observations.^8^

Table 1 summarizes the dimensions and features. It is important to note that unlike most candidate choice experiments, our experiment only features candidates who have been involved in some type of controversy. This is because our focus is on effect of the *counteraction* to that controversy on voters’ candidate choice. Only by including controversies for all candidates can we assess the effect of different types of responses on the selection of these candidates (ranging from a defense of the candidate to a costly criticism). As we show in Appendix H, it is not uncommon for politicians to be involved in the kind of controversies featured here during the course of their careers.

The first five controversies in Table 1 are examples of authoritarian behavior, based on Levitsky and Ziblatt (2018, pp. 23–24). Specifically, these features relate to (i) rejecting or only weakly committing to democratic rules (e.g. advocating that the government may ignore courts or may rule without consulting Parliament in times of crisis), (ii) denying legitimacy of political opponents (e.g. arguing that a politician from a different party constitutes a threat to Britain), (iii) tolerating or encouraging violence (e.g. encouraging online harassment of a politician from a different party), and (iv) tolerating limitations on the civil liberties of opponents (e.g. excluding journalists from press briefings). The last three controversies in Table 1 are examples of other common misdemeanors among parliamentarians, such as not responding to all messages from constituents.^9^ All controversies are selected based on real-world examples in the United Kingdom to increase the external validity of the study (see e.g., Eggers, 2014; Vivyan et al., 2012). In Appendix H, we provide real-world examples of each of the controversies and responses to controversies.

The conjoint experiment includes five different reactions to the controversial behavior (*counteractions)*. These actions can either come from multiple MPs from the candidate’s own party or multiple MPs from another party. Three reactions are counteractions, which vary in their costliness: the two most costly counteractions are the ones in which parliamentarians call for the candidate to be expelled from the parliamentary party or commit to not working with the candidate. A third, less costly, counteraction is to only criticize the candidate. Finally, the reacting MPs can defend the candidate’s behavior or not react to it.

We also incorporated other candidates’ characteristics to increase external validity. More specifically, the candidates vary by gender, party affiliation and experience, which are important characteristics that influence vote choice and are observed during campaigns (e.g., Bansak et al., 2021; Eggers et al., 2018; Schwarz & Coppock, 2022; Teele et al., 2018). Following the experimental treatment, respondents are asked questions regarding their democratic attitudes and understanding. These questions enable us to test to what extent the behaviors of the candidates are perceived as a threat to democracy.

**Table 1:** Conjoint experiment: dimensions and features

Dimensions	Features
Controversy	Argued that a politician from a different party constitutes a threat to Britain
	Argued that the government may ignore courts in times of crisis
	Argued that the government may rule without consulting Parliament in times of crisis
	Argued that the government should exclude certain journalists from press briefings
	Encouraged online harassment of a politician from a different party
	Claimed £20,000 as parliamentary expenses for private purposes
	Had an extramarital affair with a parliamentary assistant
	Ignored multiple messages from constituents
Reaction: actor	Multiple MPs from the candidate’s own party
	Multiple MPs from another party
Reaction: action	Did not react to the candidate’s behavior
	Called for the candidate to be expelled from the parliamentary party on the grounds that the candidate’s behavior was damaging to democracy
	Criticized the candidate’s behavior for damaging democracy
	Defended the candidate’s behavior
	Refused to work with the candidate on the grounds that the candidate’s behavior was damaging to democracy
Gender	Female
	Male
Party	Conservative
	Labour
Experience as minister	Yes
	No

## Results

We start our analysis of the conjoint experiment by estimating the AMCEs in line with our preregistration (Hainmueller et al., 2014). The AMCE captures the average effect of a candidate’s attribute, where the average is calculated based on all other candidate attributes. We cluster standard errors at the respondent level as respondents participate in five conjoint tasks. We show in Appendix E that there are no profile-order, carry-over or randomization effects.

**Fig. 1:**
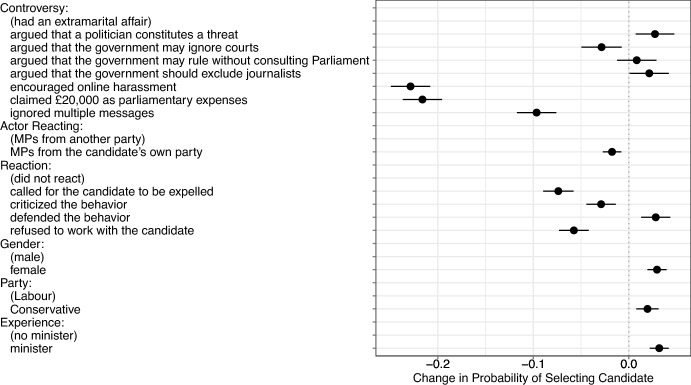
Effects of candidate features on the probability of being selected

Figure 1 summarizes the AMCEs and 95% confidence intervals as estimated using linear regression models (Hainmueller et al., 2014). The figure takes into account six dimensions of attributes: controversy, actor reacting, reaction, gender, party, experience. All estimates are computed using a baseline category written in brackets below the name of the dimension. In the following, we first examine how controversies shape vote choices and then we test our three main hypotheses, which relate to the counteractions.

### Controversies

We start analyzing the influence of controversies on respondents’ choice. As discussed above, recent experimental research has shown voters do not consistently punish politicians who display authoritarian behavior (Carey et al., 2022; Frederiksen, 2022b; Graham & Svolik, 2020).^10^ However, previous research also shows that respondents have different understandings of democracy (Grossman et al., 2022; Wunsch et al., 2023).

As Fig. 1 reveals, controversies related to authoritarian behavior do not necessarily decrease support for a candidate more strongly than other misdemeanors. More specifically, we find that the strongest effect on vote choice comes from controversies related to specific *actions* rather than *arguments*. For example, if a candidate encouraged online harassment the probability of supporting the candidate decreases by 22.9 percentage points in comparison to a candidate involved in an extramarital affair. Claiming £20,000 as parliamentary expenses reduces the probability of voting for the candidate by 21.6 percentage points. In contrast, arguing that the government may ignore courts only decreases the probability by 2.9 percentage points compared to being involved in an extramarital affair. Arguing that governments should rule without Parliament does not have a statistically significant effect on the choice and arguing that a politician constitutes a threat even increases the probability of selecting a candidate relative to an extramarital affair by 2.7 percentage points. Voters seem to perceive this authoritarian rhetoric as less concerning than actual behavior that is controversial.

To understand why respondents prefer certain forms of authoritarian behavior to other misdemeanors, we inspect how far respondents perceive the controversies as a threat to democracy. Table 2 describes how far respondents perceive the controversies as a threat for democracy on a scale from 1 to 10, where 1 means no threat and 10 serious threat. Encouraging online harassment is seen as the most serious threat (mean value: 7.4), while an extramarital affair is perceived as a relatively low threat for democracy (mean value: 4.2). We find that multiple controversies related to authoritarian behavior are not perceived as a greater threat for democracy than other misdemeanors. For example, the mean perceived threat of ignoring multiple message is 6.9, which is similar to the perceived threat of ruling without consulting Parliament. The mean perceived threat if a politician argues that a politician constitutes a threat is 5.8 and thus relatively low.

In sum, the evidence shows that controversies related to authoritarian behavior do not decrease support more strongly than other misdemeanors. This is in line with findings in recent experimental studies (Frederiksen, 2022b; Graham & Svolik, 2020; Krishnarajan, 2023).^11^ However, this raises the question at the heart of this article, namely whether other politicians’ responses can make a difference to the appeal of such politicians.

**Table 2:** Perceived threat to democracy of controversies

Controversy	Mean	SD
Argued that a politician from a different party constitutes a threat to Britain	5.83	2.37
Argued that the government may ignore courts in times of crisis	7.18	2.20
Argued that the government may rule without consulting parliament in times of crisis	6.90	2.29
Argued that the government should exclude certain journalists from press briefings	6.63	2.37
Encouraged online harassment of a politician from a different party	7.38	2.23
Claimed £20,000 as parliamentary expenses for private purposes	6.58	2.58
Had an extramarital affair with a parliamentary assistant	4.24	2.71
Ignored multiple messages from constituents	6.93	2.19

### Counteractions

We continue our analysis by examining the influence of counteractions in response to these controversies. Figure 1 summarizes the AMCEs on the actor and the action. We focus on the AMCEs as the effects of the counteractions are not systematically different for authoritarian behavior and other misdemeanors (see Appendix B).

We find strong support for the argument that counteractions have a significant effect on reducing the appeal of politicians involved in controversies, in line with hypothesis 1. If MPs criticized the behavior, called for the candidate to be expelled to refused to work with the candidate, the probability of choosing a candidate decreases. The effect sizes range from 2.9 to 7.4 percentage points. In contrast, defending the candidate increases the probability of selection by 2.8 percentage points.

Furthermore, in support of hypothesis 2, the results show that the probability of selecting a candidate decreases if the MPs reacting to the candidate comes from the candidate’s own party. More specifically, the probability decreases by 1.8 percentage points in comparison to a scenario where the actor comes from a different party. This evidence supports our theoretical expectation that counteractions coming from the same party are more credible, and thus effective than those directed at a politician from another party. In comparison to other features, the magnitude of the effect is small. If multiple MPs react to a controversy, the perceived costs appear to be limited.

Inspecting the AMCEs for each of the counteractions, we find in line with our third hypothesis that the probability of selecting a candidate decreases more when respondents are presented with counteractions that involve higher costs to the messenger. The reference category is “no reaction”. If a politician called for the candidate to be expelled the probability of selecting the candidate decreases by 7.4 percentage points. Similarly, refusing to work with a candidate reduces the probability by 5.8 percentage points compared to a scenario with no reaction. These effects are large in comparison to the effects of other candidate features such as gender, experience or various controversies. In contrast, a less costly critique of the candidate only decreases the probability by 2.9 percentage points.

**Fig. 2:**
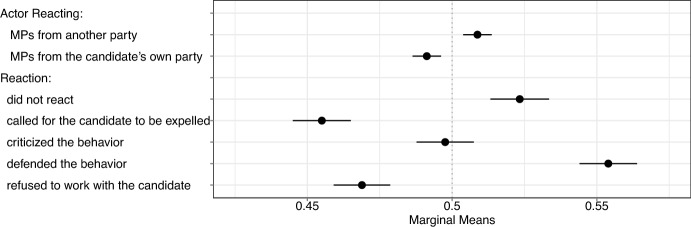
Probabilities of selecting a candidate given specific features

To illustrate the effect of the counteractions, we show the marginal means of the features related to the counteractions in Fig. 2. The marginal means capture how much respondents favor a candidate profile with a specific feature. In a forced-choice conjoint experiment with two alternatives, the marginal means correspond to the probability that respondents select a profile with that feature. Given the forced-choice design, the grand mean is 0.5, indicated by the vertical dashed line. A marginal mean of 0.5 means that the feature does not affect the choice. When a marginal mean exceeds 0.5, respondents favor profiles with that feature more often than not, and when a marginal mean is below 0.5, respondents oppose profiles with that feature more often than not. We also test whether the differences of the marginal means are statistically significant (Leeper et al., 2020).

Figure 2 summarizes these results. The probability of selecting a candidate given the actor reacting is from another party is 0.51, while the probability of selecting a candidate is 0.49. We find that the marginal means of costly counteractions are significantly lower compared to other reactions. The marginal mean decreases to 0.46 if the MPs called for the candidate to be expelled and 0.47 if they refused to work with the candidate. In contrast, the marginal mean is 0.50 if the MPs only criticized the candidate. Moreover, if other politicians actively defended the candidate—or even when they simply ignored the controversy—the candidate is viewed more favorably by voters.

In line with our preregistration and hypotheses, we focus on the average effects of counteractions but we also document variation across electoral scenarios. In Appendix D, we conduct additional analyses to assess how far the effects presented in this section hold for a scenario where (i) an undemocratic co-partisan runs against a democratic out-partisan engaged in a misdemeanor and (ii) an undemocratic co-partisan runs against a democratic co-partisan engaged in a misdemeanor. We define co-partisan as a setting where the voter thinks of herself as a supporter of the politician’s party. In line with Graham and Svolik (2020), we focus on the fraction or percentage of binary comparisons in which the undemocratic candidate is selected. Overall, we find evidence that both reacting MPs from the candidate’s own party and high-cost counteractions reduce the percentage of voters for the undemocratic politician in scenario (i) by around 5 percentage points. In scenario (ii) we find that high-cost counteractions have a strong effect on reducing the percentage of voters selecting the undemocratic co-partisan (13 percentage points). However, we do not find a statistically significant vote share reduction if the reaction comes from MPs from the same party. A potential reason for this pattern is that reacting MPs from the same party could be perceived as less credible as politicians from the same parties are competing against each other.

In sum, the evidence supports our expectations: counteractions are effective in reducing the appeal of politicians engaged in authoritarian actions and other misdemeanors.

## Robustness Checks

We assess the robustness of our results using various tests. First, we re-analyze the data using a different dependent variable. As Appendix F shows, the main results are robust if we use a rating variable as the dependent variable. Second, we apply the model-based exploratory analysis proposed by de la Cuesta et al. (2022), which permits estimating the population AMCEs using a different distribution of features (see Appendix G). Our results are robust. Third, we analyze the difference in marginal means by different subgroups.^12^ The marginal means capture how much respondents favor a candidate feature. Hence, the difference in marginal means enables us to quantify whether choices differ across subgroups. In this section, we assess whether the results differ by party identification, one of the main determinants of vote choice.

**Fig. 3:**
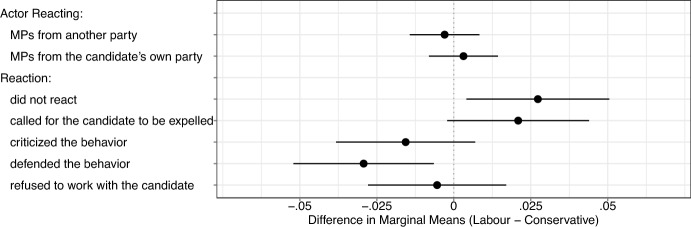
Difference in subgroup preferences by party identification

Figure 3, which illustrates the difference in marginal means by party identification, distinguishes between Labour and Conservative partisans. We focus on the theoretically relevant characteristics of the reacting actor and the counteractions. A positive difference in marginal means implies that supporters of the Labour Party tend to favor candidates with the corresponding feature more strongly than supporters of the Conservative Party. We find that the 95% confidence intervals of the differences in subgroup preferences regarding counteractions overlap with zero. This evidence suggests that high-cost actions can be an effective tool for countering authoritarian politicians, regardless of the political identification of voters.

We also explore how the results differ by pluralistic, democratic and authoritarian attitudes, which we illustrate in Appendices B and C. We measure pluralistic attitudes following Akkerman et al. (2014) and authoritarian personality using child-rearing questions following the approach of Feldman and Stenner (1997). We find some evidence that respondents with high pluralistic attitudes are less likely to choose candidates confronted with a high-cost counter. Similarly, we find that calling for the candidate to be expelled from the parliamentary party is more effective for countering individuals who attach high levels of importance to democracy. However, we do not find substantive differences in the marginal means related to the counteractions for respondents with high and low levels of authoritarian personality. However, we find that participants with higher levels of authoritarian personality are more likely to select a candidate of the Conservative Party (Engelhardt et al., 2023).

Overall, these additional results show that counteractions are effective for supporters of both major parties. Moreover, the evidence suggests that costly counteractions are most effective when respondents value democracy. This is not surprising given that these counteractions appeal directly to a potential threat against democracy.

## Conclusion

In light of the rise of populist and authoritarian politicians in advanced democracies, this article studies the effectiveness of counteractions to authoritarian behavior. Previous research has helped us understand the causes of the rise of populist and extremist politicians (Golder, 2016; Kelemen, 2017; Levitsky & Ziblatt, 2018; Mounk, 2018; Norris & Inglehart, 2019). Moreover, recent studies have shown that despite high levels of support of the principle of democracy, voters are often willing to tradeoff democratic norms in exchange for partisan victories, policy interests, security, or competence (Carey et al., 2022; Frederiksen, 2022b; Graham & Svolik, 2020; Gratton & Lee, 2024). However, we know less about what other political actors can do to counter authoritarian behavior. This question is important even in developed democracies, as it is not unusual for politicians—also within mainstream parties—to challenge democratic institutions and norms. The question is whether their fellow politician can make a difference in calling out this behavior within their ranks.

In this article, we have argued that counteractions can reduce the appeal of authoritarian politicians, and that their effectiveness depends on the credibility, and in turn the costliness, of the counteraction. The observable cost is higher when the countering actor comes from the same party as the candidate, where any criticism is unexpected and costly to both the candidate and potentially the party. Furthermore, the action’s credibility is positively related to the costliness of the action. Politicians who isolate authoritarian politicians incur higher costs as they close off valuable opportunities. For example, by publicly calling for a candidate to be expelled from the party future collaboration becomes more costly.

Our analysis supports the argument that counteractions can be an effective tool for reducing the electoral appeal of authoritarian politicians, and that the effectiveness is related to the costliness of the action. Specifically, our analysis reveals that if the messenger comes from the same party as the candidate the probability of selecting the candidate decreases by 1.8 percentage points compared to a candidate from a different party. Moreover, the costliness of the action itself matters: if the messenger calls for the candidate to be expelled from the party, the probability of selecting the candidate decreases by 7.4 percentage points in comparison to a scenario with no reaction. The effect of less costly counteractions is significantly lower.

We thus find that counteractions can make a difference to how voters view politicians who engage in authoritarian behavior. More worryingly, our findings also show that such counteractions may be necessary to reduce the appeal of authoritarian politicians. We find that voters do not consistently perceive authoritarian behavior as a greater threat to democracy than other misdemeanors. In the absence of counteractions by other politicians, voters are no more likely to reject candidates for their authoritarian behaviors, such as advocating restricting the powers of courts and parliament, than other misdemeanors, such as failing to answer constituency emails. This is in line with recent research that has shown that voters do not always punish authoritarian behavior (Carey et al., 2022; Frederiksen, 2022b; Graham & Svolik, 2020). This suggests that counteractions are not only effective, but may also be necessary in drawing voters’ attention to why certain authoritarian behaviors may constitute a greater threat to democracy than other misdemeanor of politicians. A limitation of our study is the focus on a single country. Yet, our expectation is that the findings would generalize to other established democracies, where the experimental literature has found very similar tolerance of authoritarian behavior by mainstream politicians, such as in the US (Graham & Svolik, 2020; Grillo & Prato, 2023; Wunsch et al., 2023). Our work also speaks to the role of mainstream parties defending democratic norms. The findings highlight the crucial role of politicians within in mainstream parties, such as the Republican Party in the US and the Conservative Party in the UK, in countering any authoritarian behavior they may observe within their ranks.

## Supplementary Information

Below is the link to the electronic supplementary material.

Supplementary file 1 (pdf 357 KB)

## Data Availability

The replication files are available at the Political Behavior Dataverse: 10.7910/DVN/EBSUNP.

## Footnotes

1 An example of this was Boris Johnson’s prorogation of Parliament in 2019, which the Supreme Court ruled to be unlawful (Grillo & Prato, 2023).

2 Exceptions include recent studies by Wuttke and Foos (2024), which tests the effect of ‘democratic talk’ on liberal democratic attitudes in Germany, and Voelkel et al. (2023) who show that correcting stereotypes and misperceptions, as well as outlining the consequences of democratic collapse, decreases support for authoritarian behavior in the United States.

3 We have preregistered each of the hypotheses tested in the article. In our preregistration, we specified ten hypotheses. However, the focus of this article is on the three hypotheses that deal with the effectiveness of counteractions, preregistered as hypotheses 2 (H1 in article), 4 (H2 in article) and 5 (H3 in article). The remaining hypotheses focus on the effects of authoritarian behavior on candidate choice (hypotheses 1 and 3) and subgroup effects (6–10). In Appendix B, we show and discuss the results of each of the preregistered hypotheses.

4 We focus on England since the other three nations of the UK have different party systems. England makes up more than 80% of the UK population. Deltapoll uses active sampling and re-weighting to create samples based on a panel of respondents provided by Dynata encompassing 750,000 individuals in Great Britain. Deltapoll pays a small incentive to respondents.

5 Labour and Conservative MPs won 98% of English constituencies in the 2019 General Election.

6 The preregistration can be found here: https://osf.io/zc7b2/?view_only=1820075f934c4adabd18aeaa8033842f. All preregistered hypotheses are listed and evaluated empirically in Appendix B. In the article itself, we focus on our three main hypotheses that concern the counteractions (preregistered as hypotheses 2, 4 and 5).

7 To increase readability of the candidate profiles, the dimension on the actor reacting always appears above the actor’s reaction.

8 The candidates’ features are uniformly distributed as we do not have data on their real-world distribution (de la Cuesta et al., 2022) However, we assess the sensitivity of our results given this assumption by using model-based exploratory analysis proposed by de la Cuesta et al. (2022) (see Appendix G).

9 See for example the evidence reported by My Society: https://www.mysociety.org/2014/06/10/how-responsive-is-your-mp/ (last accessed: June 26, 2024).

10 The reference category is the controversy of a extramarital affair because this is a controversy involving low levels of threat to democracy.

11 This evidence is not in line with our preregistered hypotheses 1 and 3, which assume that voters are less likely to vote for politicians displaying authoritarian behavior.

12 This analysis is connected to our preregistered hypotheses 6–10 on subgroup effects, which we test in Appendix B. We find supporting evidence for hypothesis 6, mixed evidence for hypotheses 8 and 9, and refute hypotheses 7 and 10.

## References

[CR1] Akkerman, A., Mudde, C., & Zaslove, A. (2014). How populist are the people? Measuring populist attitudes in voters. *Comparative Political Studies,**47*(9), 1324–1353.

[CR2] Andreottola, G. (2021). Flip-flopping and electoral concerns. *Journal of Politics,**83*(4), 1669–1680.

[CR3] Bansak, K., Hainmueller, J., Hopkins, D. J., & Yamamoto, T. (2021). Conjoint survey experiments. In J. N. Druckman & D. P. Green (Eds.), *Advances in experimental political science* (pp. 19–41). Cambridge University Press.

[CR4] Bansak, K., Hainmueller, J., Hopkins, D. J., & Yamamoto, T. (2023). Using conjoint experiments to analyze election outcomes: The essential role of the average marginal component effect. *Political Analysis,**31*(4), 500–518.

[CR5] Becher, M., & Brouard, S. (2022). Executive accountability beyond outcomes: Experimental evidence on public evaluations of powerful prime ministers. *American Journal of Political Science,**66*(1), 106–122.

[CR6] Bellamy, R., & Kröger, S. (2021). Countering democratic backsliding by EU member states: Constitutional pluralism and ‘value’ differentiated integration. *Swiss Political Science Review,**27*(3), 619–636.

[CR7] Berinsky, A. J. (2017). Rumors and health care reform: Experiments in political misinformation. *British Journal of Political Science,**47*(2), 241–261.

[CR8] Besley, T. (2006). *Principled agents? The political economy of good government*. Oxford University Press.

[CR9] Betsy, N., & Goldsmith, T. (2019). *How parliament works*. Routledge.

[CR10] Bor, A., Mazepus, H., Bokemper, S. E., & DeScioli, P. (2021). When should the majority rule? Experimental evidence for Madisonian judgments in five cultures. *Journal of Experimental Political Science,**8*(1), 41–50.

[CR11] Burgoon, B., van Noort, S., Rooduijn, M., & Underhill, G. (2019). Positional deprivation and support for radical right and radical left parties. *Economic Policy,**34*(97), 49–93.

[CR12] Carey, J. M. (2007). Competing principals, political institutions, and party unity in legislative voting. *American Journal of Political Science,**51*(1), 92–107.

[CR13] Carey, J. M., Clayton, K., Helmke, G., Nyhan, B., Sanders, M., & Stokes, S. (2022). Who will defend democracy? Evaluating tradeoffs in candidate support among partisan donors and voters. *Journal of Elections, Public Opinion and Parties,**32*(1), 230–245.

[CR14] Chiang, C.-F., & Knight, B. (2011). Media bias and influence: Evidence from newspaper endorsements. *Review of Economic Studies,**78*(3), 795–820.

[CR15] Cho, I.-K., & Kreps, D. M. (1987). Signaling games and stable equilibria. *Quarterly Journal of Economics,**102*(2), 179–221.

[CR16] Chong, D. (1993). How people think, reason, and feel about rights and liberties. *American Journal of Political Science,**37*(3), 867–899.

[CR17] Clayton, K., & Willer, R. (2023). Endorsements from Republican politicians can increase confidence in US elections. *Research & Politics,**10*(1), 1–5.

[CR18] Cukierman, A., & Tommasi, M. (1998). When does it take a Nixon to go to China? *American Economic Review,**88*(1), 180–197.

[CR20] Dahl, R. (1989). *Democracy and its critics*. Yale University Press.

[CR19] Dahl, R. A. (1961). *Who governs? Democracy and power in an American City*. Yale University Press.

[CR21] Dalton, R. J., Sin, T., & Jou, W. (2007). Understanding democracy: Data from unlikely places. *Journal of Democracy,**18*(4), 142–156.

[CR22] de la Cuesta, B., Egami, N., & Imai, K. (2022). Improving the external validity of conjoint analysis: The essential role of profile distribution. *Political Analysis,**30*(1), 19–45.

[CR23] De Vries, C. E., & Hobolt, S. (2020). *Political entrepreneurs: The rise of challenger parties in Europe*. Princeton University Press.

[CR24] Denver, D., & Johns, R. (2022). *Elections and voters in Britain*. Palgrave Macmillan.

[CR25] Devine, D. (2023). Is support for liberal democracy in crisis? *Political Insight,**14*(1), 8–11.

[CR26] Donovan, T. (2019). Authoritarian attitudes and support for radical right populists. *Journal of Elections, Public Opinion and Parties,**29*(4), 448–464.

[CR27] Druckman, J. N. (2001). On the limits of framing effects: Who can frame? *Journal of Politics,**63*(4), 1041–1066.

[CR28] Eggers, A. C. (2014). Partisanship and electoral accountability: Evidence from the UK expenses scandal. *Quarterly Journal of Political Science,**9*(4), 441–472.

[CR29] Eggers, A. C., Vivyan, N., & Wagner, M. (2018). Corruption, accountability, and gender: Do female politicians face higher standards in public life? *Journal of Politics,**80*(1), 321–326.

[CR30] Engelhardt, A. M., Feldman, S., & Hetherington, M. J. (2023). Advancing the measurement of authoritarianism. *Political Behavior,**45*, 537–560.

[CR31] Fearon, J. D. (1994). Domestic political audiences and the escalation of international disputes. *American Political Science Review,**88*(3), 577–592.

[CR32] Feldman, S., & Stenner, K. (1997). Perceived threat and authoritarianism. *Political Psychology,**18*(4), 741–770.

[CR33] Frederiksen, K. V. S. (2022a). Do two-party systems hamper defection from undemocratic candidates? https://osf.io/preprints/osf/wmzj3

[CR34] Frederiksen, K. V. S. (2022b). Does competence make citizens tolerate undemocratic behavior? *American Political Science Review,**116*(3), 1147–1153.

[CR35] Frederiksen, K. V. S. (2022c). When democratic experience distorts democracy: Citizen reactions to undemocratic incumbent behaviour. *European Journal of Political Research,**61*(1), 281–292.

[CR36] Gambetta, D. (2009). Signaling. In P. Bearman & P. Hedström (Eds.), *The Oxford handbook of analytical sociology* (pp. 168–194). Oxford University Press.

[CR37] Golder, M. (2016). Far right parties in Europe. *Annual Review of Political Science,**19*, 477–497.

[CR38] Graham, M., & Svolik, M. W. (2020). Democracy in America? Partisanship, polarization, and the robustness of support for democracy in the United States. *American Political Science Review,**114*(2), 392–409.

[CR39] Gratton, G., & Lee, B. E. (2024). Liberty, security, and accountability: The rise and fall of illiberal democracies. *The Review of Economic Studies,**91*, 340–371.

[CR40] Greene, J., & Jennings, W. (2017). *The politics of competence*. Cambridge University Press.

[CR41] Grillo, E., & Prato, C. (2023). Reference points and democratic backsliding. *American Journal of Political Science,**67*(1), 71–88.

[CR42] Grossman, G., Kronick, D., Levendusky, M., & Meredith, M. (2022). The majoritarian threat to liberal democracy. *Journal of Experimental Political Science,**9*(1), 36–45.

[CR43] Hahm, H., König, T., Osnabrügge, M., & Frech, E. (2019). Who settles disputes? Treaty design and trade attitudes toward the transatlantic trade and investment partnership. *International Organization,**73*(4), 881–900.

[CR44] Hainmueller, J., Hopkins, D., & Yamamoto, T. (2014). Causal inference in conjoint analysis: Understanding multidimensional choices via stated preference experiments. *Political Analysis,**22*(1), 1–30.

[CR45] Horiuchi, Y., Markovich, Z., & Yamamoto, T. (2022). Does conjoint analysis mitigate social desirability bias? *Political Analysis,**30*(4), 535–549.

[CR46] Hovland, C. I., Janis, I. L., & Kelley, H. H. (1953). *Communication and persuasion: Psychological studies of opinion changes*. Yale University Press.

[CR47] Inglehart, R., & Norris, P. (2003). *Rising tide: Gender equality and cultural change around the world*. Cambridge University Press.

[CR48] Kelemen, R. D. (2017). Europe’s other democratic deficit: National authoritarianism in Europe’s Democratic Union. *Government and Opposition,**52*(2), 211–238.

[CR49] Klingemann, H.-D. (2014). Dissatisfied democrats: Democratic maturation in old and new democracies. In R. J. Dalton & C. Welzel (Eds.), *The civic culture transformed: From allegiant to assertive citizens* (pp. 116–157). Cambridge University Press.

[CR50] Krishnarajan, S. (2023). Rationalizing democracy: The perceptual bias and (un)democratic behavior. *American Political Science Review,**117*(2), 474–496.

[CR51] Leeper, T., Hobolt, S., & Tilley, J. (2020). Measuring subgroup preferences in conjoint experiments. *Political Analysis,**28*(2), 207–221.

[CR52] Levitsky, S., & Ziblatt, D. (2018). *How democracies die*. Penguin Random House.

[CR53] Linz, J. (2000). *Totalitarian and authoritarian regimes*. Lynne Rienner.

[CR54] Lupia, A., & McCubbins, M. D. (1998). *The democratic dilemma: Can citizens learn what the need to know?* Cambridge University Press.

[CR55] Lührmann, A. (2021). Disrupting the autocratization sequence: Towards democratic resilience. *Democratization,**28*(5), 1017–1039.

[CR56] Mounk, Y. (2018). *The people vs. democracy. Why our freedom is in danger & how to save it*. Harvard University Press.

[CR57] Norris, P., & Inglehart, R. (2019). *Cultural backlash: Trump, Brexit, and authoritarian populism*. Cambridge University Press.

[CR58] Norton, P. (2013). *Parliament in British politics*. Palgrave Macmillan.

[CR59] Proctor, K. (2019). Boris Johnson’s move to prorogue Parliament ‘a constitutional outrage’, Says Speaker. https://www.theguardian.com/politics/2019/aug/28/boris-johnsons-move-to-prorogue-parliament-a-constitutional-outrage-says-speaker

[CR60] Proksch, S.-O., & Slapin, J. B. (2012). Institutional foundations of legislative speech. *American Journal of Political Science,**56*(3), 520–537.

[CR61] Przeworski, A. (2019). *Crises of democracy*. Cambridge University Press.

[CR62] Robinson, T. S. (2023). When do voters respond to campaign finance disclosure? Evidence from multiple election types. *Political Behavior,**45*, 1309–1332.

[CR63] Schwarz, S., & Coppock, A. (2022). What have we learned about gender from candidate choice experiments? A meta-analysis of sixty-seven factorial survey experiments. *Journal of Politics,**84*(2), 655–668.

[CR64] Somer, M., McCoy, J. L., & Luke, R. E. (2021). Pernicious polarization, autocratization and opposition strategies. *Democratization,**28*(5), 929–948.

[CR65] Spence, M. (1973). Job market signaling. *Quarterly Journal of Economics,**87*(3), 355–374.

[CR66] Teele, D. L., Kalla, J., & Rosebluth, F. (2018). The ties that double bind: Social roles and women’s underrepresentation in politics. *American Political Science Review,**112*(3), 525–541.

[CR67] Vivyan, N., Wagner, M., & Tarlov, J. (2012). Representative misconduct, voter perceptions and accountability: Evidence from the 2009 House of Commons Expenses Scandal. *Electoral Studies,**31*(4), 750–763. 23576832 10.1016/j.electstud.2012.06.010 PMC3617916

[CR68] Voelkel, J. G., Stagnaro, M. N., Chu, J., Pink, S. L., Mernyk, J. S., Redekoop, C., Ghezae, I., Cashman, M., Adjodah, D., Allen, L., Allis, V., Baleria, G., Ballantyne, N., Van Bavel, J. J., Blunden, H., Braley, A., Bryan, C., Celniker, J., Cikara, M., ... Willer, R. (2023). Megastudy identifying effective interventions to strengthen Americans’ democratic attitudes. Working Paper.

[CR69] Wagner, M., Glinitzer, K., & Vivyan, N. (2020). Costly signals: Voter responses to parliamentary dissent in Austria, Britain, and Germany. *Legislative Studies Quarterly,**45*(4), 645–678.

[CR70] Weingast, B. R. (1997). The political foundations of democracy and the rule of the law. *American Political Science Review,**91*(2), 245–263.

[CR71] Wunsch, N., Jacob, M. S., & Derksen, L. (2023). The demand side of democratic backsliding: How divergent understandings of democracy shape political choice. Preprint.

[CR72] Wuttke, A., & Foos, F. (2024). Making the case for democracy: A field-experiment on democratic persuasion. *European Journal of Political Research*. 10.1111/1475-6765.12705

[CR73] Wuttke, A., Gavras, K., & Schoen, H. (2022). Have European grown tired of democracy? New evidence from 18 consolidated democracies, 1981–2018. *British Journal of Political Science,**52*(1), 416–428.

[CR74] Zakaria, F. (1997). The rise of illiberal democracy. *Foreign Affairs,**76*, 22–43.

