# Supplementary material for: Countering Authoritarian Behavior in Democracies
Source: Polit Behav. 2024 Sep 13;47(2):781–800. doi: 10.1007/s11109-024-09971-5 (PMC12052917; doi:10.1007/s11109-024-09971-5)
Supplement: Supplementary file 1 — (pdf 357 KB) [file 11109_2024_9971_MOESM1_ESM.pdf]

Appendices
Countering Authoritarian Behavior in Democracies

Sara B. Hobolt
London School of Economics
s.b.hobolt@lse.ac.uk

Moritz Osnabrügge
Durham University
moritz.osnabruegge@durham.ac.uk

Contents

A Representativeness of the Sample **1**

B Preregistration **2**

B.1 Authoritarian Behavior 4

B.2 Subgroup Analysis 6

C Further Results **9**

D Analysis of Two Electoral Scenarios **11**

E Assumptions **12**

F Alternative Dependent Variable **16**

G Alternative Distribution of Candidate Features **17**

H Examples of Controversies and Counteractions **19**

H.1 Controversies 19

H.2 Counteractions 21

I Questionnaire **21**

I.1 Pre-Treatment 21

I.2 Survey Experiment 23

I.3 Post-Treatment 25

A Representativeness of the Sample

We implemented our survey using Deltapoll[1], which uses active sampling to ensure that the sample is nationally representative. Deltapoll collaborates with Dynata and has access to a panel of 750,000 individuals in the United Kingdom. Participants receive a small incentive for their participation. Table A1 compares our sample to the 2011 UK Census focusing on adult individuals (18 years and older). The table summarizes the proportion of individuals by age group, gender and region. The table shows that the distributions in the population and our sample are very similar.[2]

Table A1: Representativeness of the Sample

Variable	Feature	Census	Sample
Age	18-24	0.12	0.12
	25-34	0.17	0.17
	35-44	0.18	0.16
	45-54	0.17	0.18
	55-64	0.15	0.15
	65+	0.21	0.22
Gender	Female	0.51	0.51
Region	North West	0.13	0.13
	North East	0.05	0.05
	Yorkshire and the Humber	0.10	0.10
	Eastern	0.11	0.11
	West Midlands	0.10	0.10
	East Midlands	0.09	0.08
	South East	0.16	0.17
	London	0.15	0.16
	South West	0.10	0.10

[1] More details on Deltapoll can be found here: `https://deltapoll.co.uk/` (last accessed: August 26, 2022).

[2] The Census data was collected in 2011, which was the most recent Census at the time of data collection.

B Preregistration

As outlined in the paper, we preregistered our experiment at https://osf.io/zc7b2/?view_only=1820075f934c4adabd18aeaa8033842f. We *use the terms authoritarian behavior and illiberal behavior as synonyms.* We specified ten hypotheses but our main focus lies on hypotheses 2, 4, and 5 as we are interested in the effectiveness of counteractions. Hypothesis 2 is the first hypothesis in our paper. Hypothesis 4 corresponds to the second hypothesis presented in the paper and hypothesis 5 is equal to the third hypothesis. The other hypotheses reported in the preregistration report are aimed at providing additional results. More specifically, hypotheses 1 and 3 examine the effect of authoritarian behavior, and hypotheses 6-10 focus on subgroup effects.

Authoritarian Behavior and Counteractions:

> Hypothesis 1: Respondents are less likely to prefer candidates who have been involved in controversies that may be seen to go against democratic norms ("illiberal behavior") than candidates involved in general misdemeanors.
>
> Hypothesis 2: Respondents are less likely to prefer a candidate who has been isolated or whose views or actions have been criticized by other politicians ("counteraction") than candidates who have not been criticized.
>
> Hypothesis 3: The effect of the counteraction is greater for illiberal behavior than general misdemeanors.
>
> Hypothesis 4: The effect of the counteraction is greater when the politicians engaging in the counteraction belong to the same party as the candidate than when they belong to another party.

Hypothesis 5: The more costly the counteraction, the greater the effect on respondents' likelihood of preferring a candidate.

Subgroup Analysis:

Hypothesis 6: Respondents are more likely to support a candidate involved in controversy if they belong to a party they normally support than if they belong to a party they don't normally support.

Hypothesis 7: Respondents who support the party of the politicians engaging in the counteraction (in-group party counteraction) are less likely to prefer the candidate who is met with a counteraction compared with respondents who do not support the party of the politicians engaging in the counteraction (out-group party counteraction).

Hypothesis 8: Respondents who value pluralism more are less likely to prefer a candidate who is met with a counteraction than respondents who value pluralism less.

Hypothesis 9: Respondents who have a more authoritarian personality are more likely to prefer candidates engaging in illiberal behavior than those with a less authoritarian personality.

Hypothesis 10: Respondents who have a more authoritarian personality are more likely to prefer a candidate who is met with a counteraction than respondents with a less authoritarian personality.

In the following, we discuss the results when testing each of these hypotheses. In the paper, we focus on our three main hypotheses related to the counteractions (hypotheses 2, 4, and 5). This section presents results on the other hypotheses.

B.1 Authoritarian Behavior

The evidence does not support hypothesis 1, as respondents are not more likely to select candidates involved in misdemeanors than authoritarian behavior. Figure 1 in the manuscript summarizes the Average Marginal Component Effects (AMCEs). For example, the strongest negative effect refers to the AMCE on using parliamentary expenses for private purposes, which is a misdemeanor. This evidence is in line with previous research showing that voters sometimes tolerate authoritarian behavior (Frederiksen, 2022; Graham and Svolik, 2020; Krishnarajan, 2023).

Hypothesis 3 expects that the counteraction's effect is stronger for authoritarian behavior than for general misdemeanors. This hypothesis assumes that respondents perceive authoritarian behavior as a greater threat to democracy than other misdemeanors. We do not find evidence in line with this hypothesis as this assumption is unmet (see evidence on hypothesis 1). In Figure A1, we present the interaction effects between the controversy and the counteractions. Technically, we estimate the Average Component Interaction Effects (ACIEs) using linear regression models (Hainmueller, Hopkins and Yamamoto, 2014). As the figure illustrates, we find that most of the effects are statistically not significant. Also, the visual inspection suggests that the interaction effects do not differ systematically between authoritarian behavior and general misdemeanors. We also calculated Average Marginal Interaction Effects (AMIEs) as a robustness test and found consistent results (Egami and Imai, 2019). These results suggest that high-cost actions effectively counter undemocratic behavior and misdemeanors.

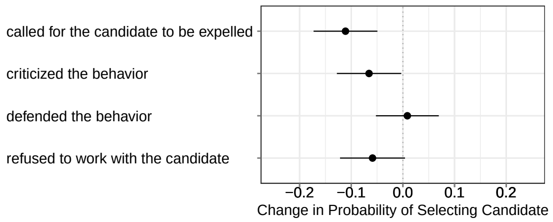

(a) Argued that a politician constitutes a threat

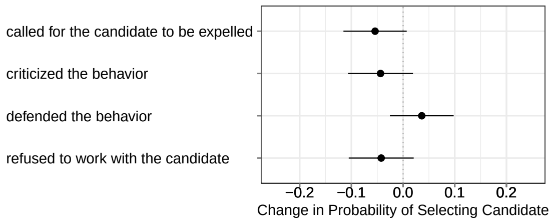

(b) Argued that the government may ignore courts

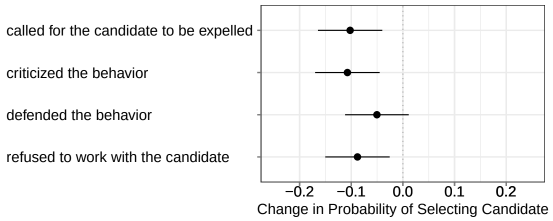

(c) Argued that the government may rule without consulting Parliament

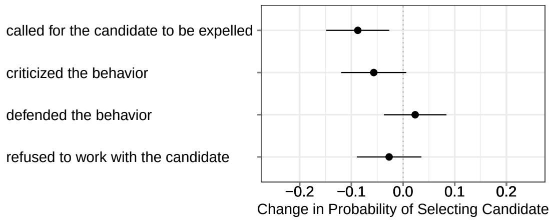

(d) Argued that the government should exclude journalists

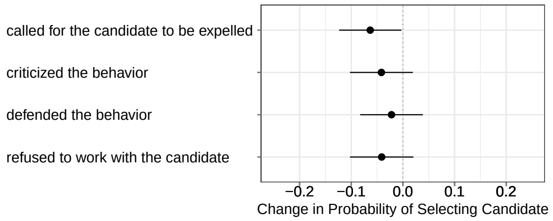

(e) Claimed £20,000 as parliamentary expenses

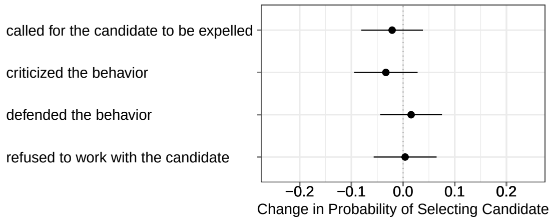

(f) Encouraged online harassment

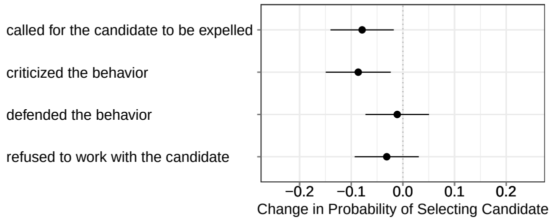

(g) Ignored multiple messages

Figure A1: Interaction Effects between Controversies and Counteractions

Note: ACIEs and 95% confidence intervals are computed using an OLS regression model.

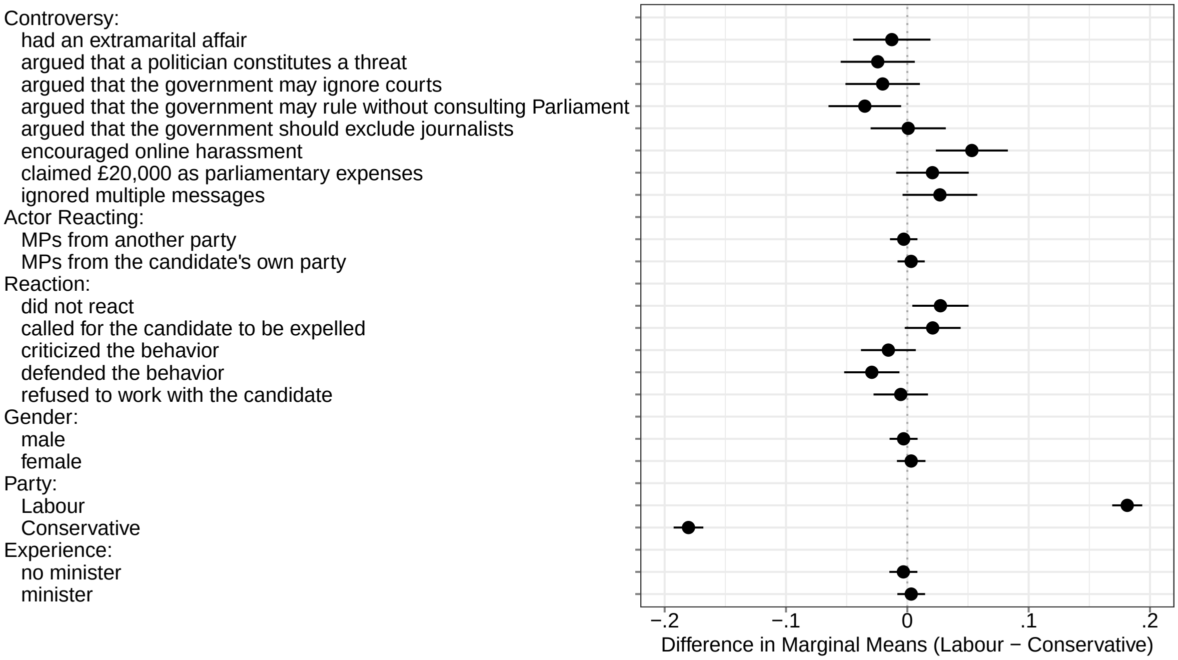

Figure A2: Difference in Subgroup Preferences by Party

B.2 Subgroup Analysis

In line with the preregistration, we focus on the difference in marginal means when analyzing subgroup preferences. We start with inspecting hypothesis 6, which is corroborated by the evidence as the respondents supporting a specific party are more likely to select a candidate from this party. Figure A2 illustrates the difference in subgroup preferences of participants who see themselves closer to the Conservative Party and participants who see themselves closer to the Labour Party. It illustrates differences in marginal means and 95% confidence intervals computed following Leeper, Hobolt and Tilley (2020). As the figure demonstrates, party supporters are more likely to vote for a candidate from the party they usually support.

To test hypothesis 7, we estimate interaction effects between the candidate's party and the actor reacting to the candidate. More specifically, we calculate ACIEs and AMIEs for supporters of the Conservative and Labour parties (Egami and Imai, 2019).

As we do not find statistically significant interaction effects, we refute hypothesis 7.

Hypothesis 8 is tested by examining the difference in marginal means of respondents with high levels of pluralism and respondents with low levels of pluralism. We measure pluralism by using the methodology of Akkerman, Mudde and Zaslove (2014). Figure A3 shows the results. We find that respondents with pluralistic attitudes exhibit a more negative reaction to a situation where an actor refused to work with the candidate. The 95% confidence interval of the difference in marginal means does not overlap with zero. However, we do not find this pattern if the actor calls for the candidate to be expelled. Hence, we find mixed evidence for hypothesis 8.

We investigate hypotheses 9 and 10 by examining the difference in marginal means of respondents with high and low levels of authoritarian personality, which is measured using child-rearing questions (e.g., Feldman and Stenner, 1997; Stenner, 2005). Figure A4 illustrates the difference in marginal means by respondents with high and low levels of authoritarianism. We do not find consistent evidence that subgroup preferences differ. The finding on the controversies (hypothesis 9) makes sense as participants did not distinguish between authoritarian behavior and general misdemeanors. Next, we study the marginal means related to the counteractions. The direction of the coefficients in Figure A4 appear consistent with the hypothesis as the difference in marginal means is positive. However, the differences are not substantive as the 95% confidence interval of the difference in marginal means overlaps with zero. Hence, we refute hypothesis 10. One potential reason for this finding refers to the measure of authoritarianism, which focuses on child-rearing attitudes, an indirect measure of authoritarian attitudes.

.

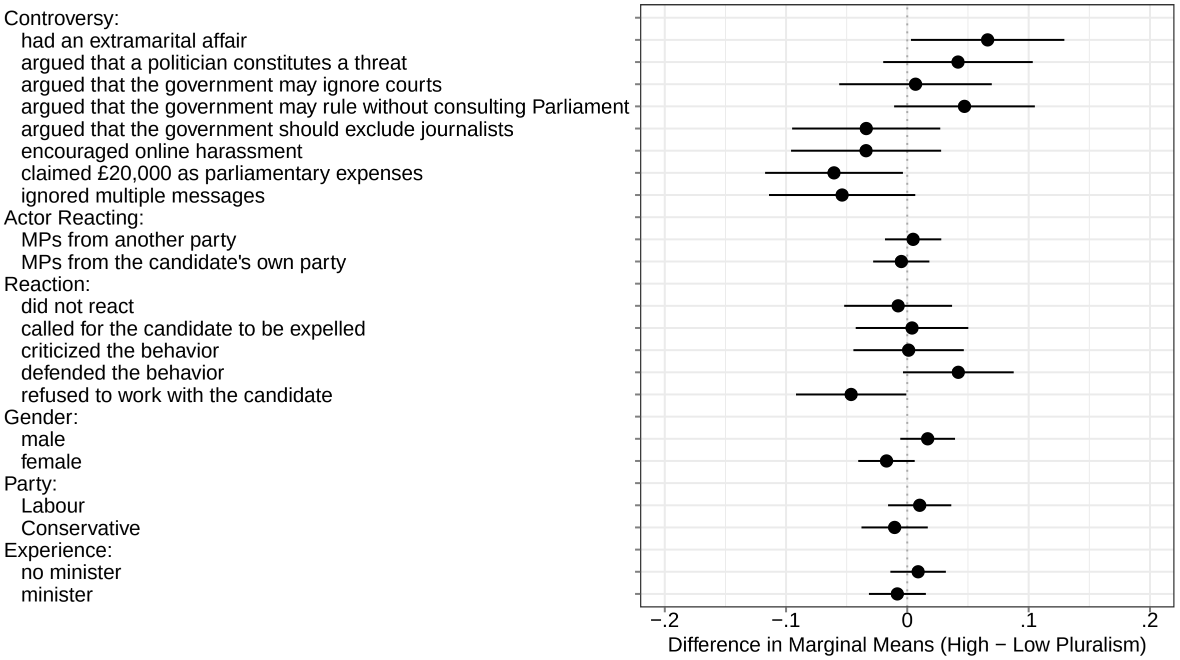

Figure A3: Difference in Subgroup Preferences by Pluralism

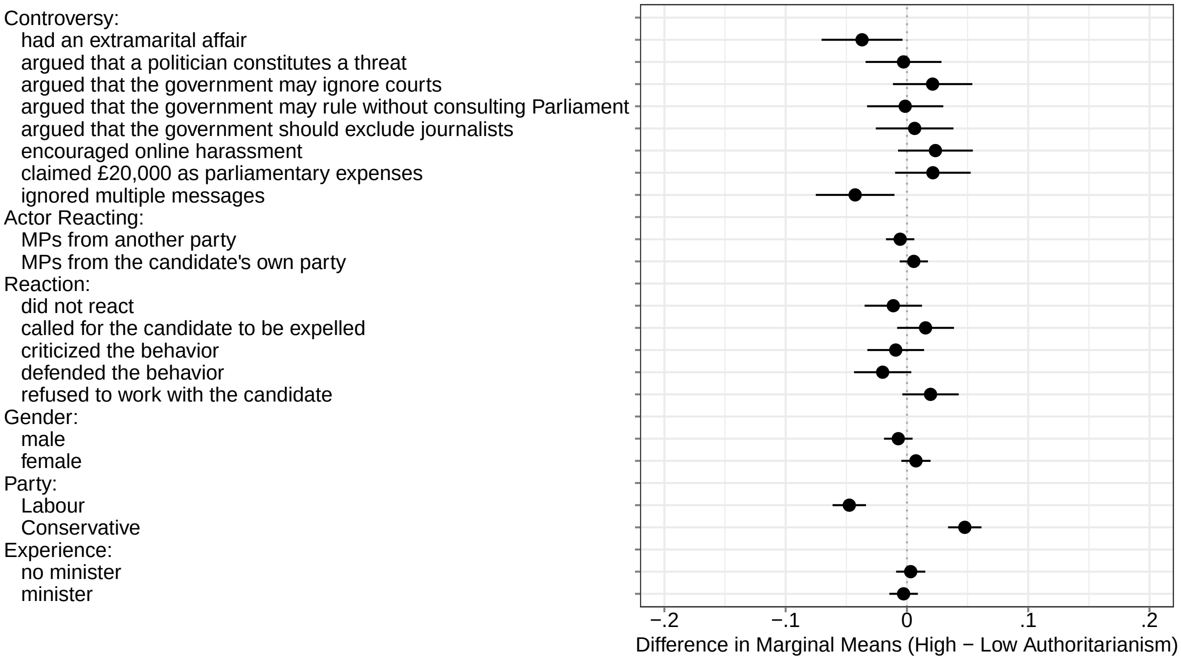

Figure A4: Difference in Subgroup Preferences by Authoritarian Personality

C Further Results

We investigate to what degree candidate preferences differ by attitudes toward democracy. We examine the difference in marginal means by respondents who think living in a democracy is very important or not very important. Figure A5 summarizes our results. Regarding the reactions, our main finding is that the costliness of the counteraction has a greater influence on respondents who attach high importance to democracy. Respondents who think that living in a democracy is important are less likely to choose a candidate if MPs called for the candidate to be expelled (probability: 0.44). Respondents who think that democracy is less important do not react that strongly to this costly signal. The 95% confidence interval on the difference in marginal means does not overlap with zero. Graphically, we also observe that subgroup preferences differ when parliamentarians refuse to work with a candidate but these differences are not statistically significant.

We also analyze interaction effects between the features on the reacting actor and the specific action. This procedure allows disentangling how far the effect of the actions differs by the party affiliation of the countering politicians. Figure A6 summarizes the ACIEs, which we estimate using a linear regression model. The estimates capture the additional effect of counteractions given the MPs are from the candidate's own party. As the figure shows, we do not find statistically significant interaction effects between the features related to the actor and the action. The evidence underlines that party affiliation does not only have implications for the credibility of counteractions but also for supportive and neutral actions. We also computed the AMIEs based on ANOVA regression and got the same results (Egami and Imai, 2019).

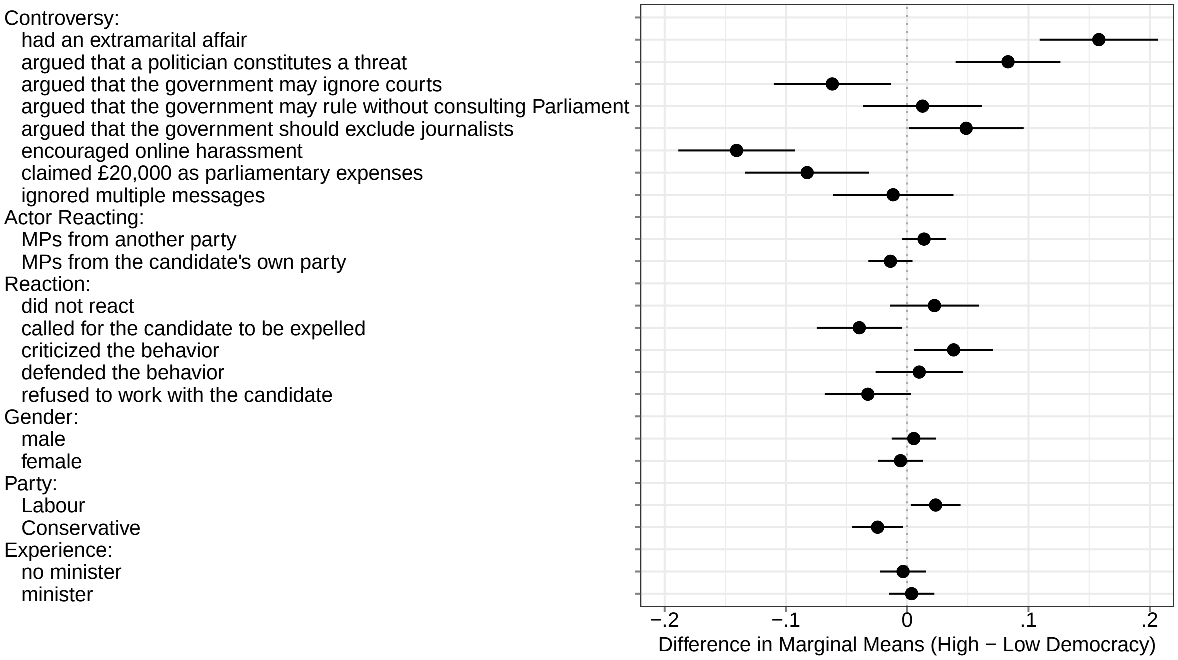

Figure A5: Difference in Subgroup Preferences by Importance Attached to Democracy

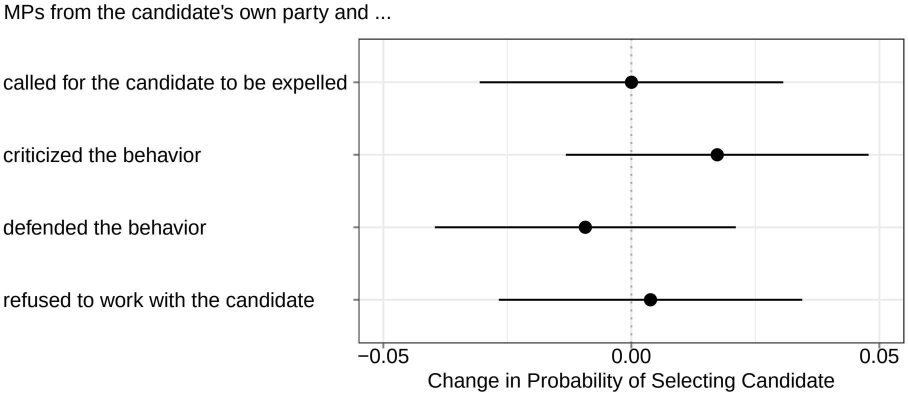

Figure A6: ACIEs for attributes related to the reacting actor and the action.

Note: The baseline categories are the features “MPs from another party” and “did not react”.

D Analysis of Two Electoral Scenarios

In line with our hypotheses and research design our main focus lies on the average effects of counteractions. However, our experimental design also allows us to analyze specific scenarios. In the following, we analyze scenarios where (i) an undemocratic co-partisan runs against a democratic out-partisan engaged in a misdemeanor and (ii) an undemocratic co-partisan runs against a democratic co-partisan engaged in a misdemeanor. We define co-partisan as a setting where the voter thinks of herself as a supporter of the politician's party. In line with Graham and Svolik (2020), we focus on the fraction or percentage of binary comparisons in which the undemocratic candidate is selected.

We start inspecting scenario (i) where we find that 73% of the participants vote for the undemocratic in-partisan candidate. Next, we continue exploring the percentage of participants voting for the undemocratic candidate given different reacting actors and counteractions. When the reacting actors are multiple parliamentarians from the candidate's party, the percentage of co-partisans choosing an undemocratic candidate is reduced to 70%. On the other hand, the percentage corresponds to 75% if the countering actors are parliamentarians from the opposing party. This difference in proportions is statistically significant at the 0.05 level. Examining the type of counteraction, the percentage of voters selecting the undemocratic politician when faced with a high-cost counteraction is reduced to 70% while it is 75% when facing a different reaction. This difference is also statistically significant at the 0.05 level. Overall, we find that the effectiveness of the type of actor and the type of action are similar in scenario (i). Both the in-group actor and the high-cost counteractions reduce the percentage of participants voting for the undemocratic politician by around 5 percentage points.

In scenario (ii) we find that voters select the undemocratic politician in 60% of

the choice tasks. This is, unsurprisingly, much lower than in scenario (i). If the actor countering the candidate comes from the same party, this percentage of participants selecting the undemocratic candidate decreases to 58%, while the percentage is 61% if the countering politician comes from a different party. The difference between these two percentages is not statistically significant at the 0.05 level. We continue by analyzing the choices when facing candidates with different types of counteractions. If a candidate receives a high-cost counteraction, the percentage of voters selecting the undemocratic co-partisan is 52%, while the percentage is 65% when facing a low-cost counteraction. This difference corresponds to 13 percentage points and is statistically significant.

The analysis reveals two interesting findings. First, the type of action is especially effective in scenarios where two candidates from the same party compete against each other (scenario ii). Second, the type of actor has a stronger influence on the choice in the scenario (i) rather than (ii).

E Assumptions

We check the assumptions made to estimate the AMCEs and ACIEs (Hainmueller, Hopkins and Yamamoto, 2014). First, we investigate whether profile-order effects exist. In our experiment, respondents are shown a profile on the left and right sides. To examine the assumption that no profile-order effects exist, we create a subsample of observations related to the left and right panel and estimate the AMCEs for each of the samples. Figure A7 shows the results and provides evidence that the effects do not differ substantially across panels. We only observe minor differences. For example, the controversy on ignoring courts exhibits a negative AMCE on the right panel, but the AMCE of the left panel overlaps with zero. The findings on the counteractions are robust.

We continue examining potential carry-over effects. The experiment presents respondents five times two profiles. We thus divide the sample in a subsample related to each round and estimate the AMCEs based on these subsamples. As Figure A8 shows, the AMCEs are very similar across rounds, which rules out concerns regarding profile order effects.

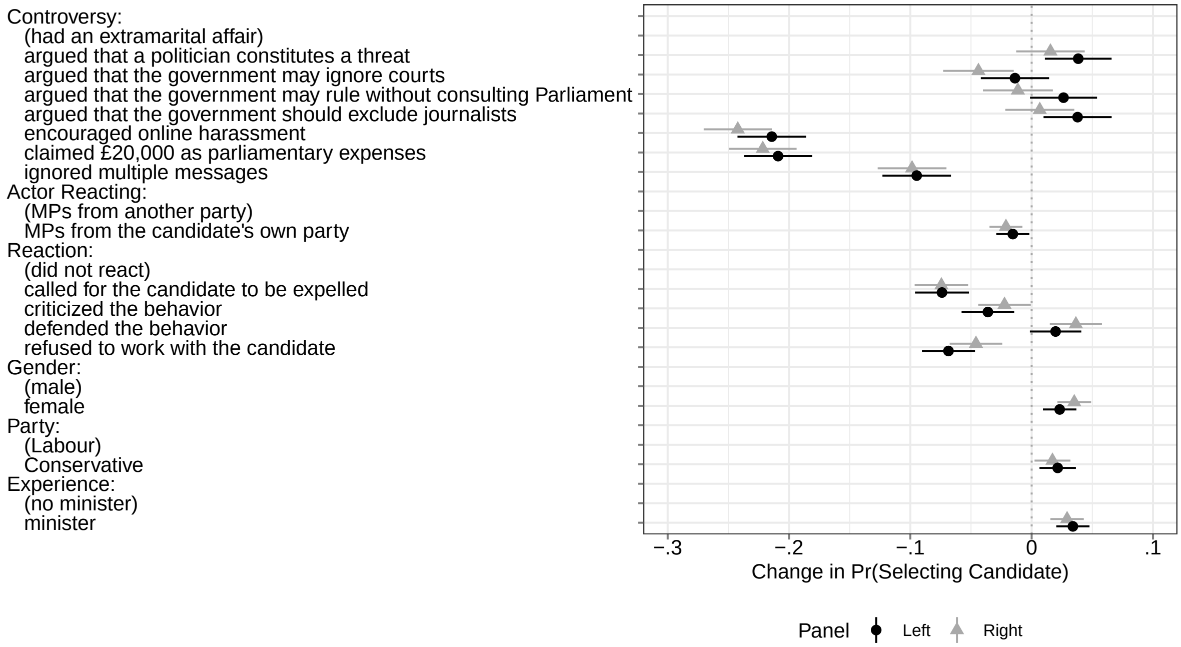

Figure A7: Conditional AMCEs by panel

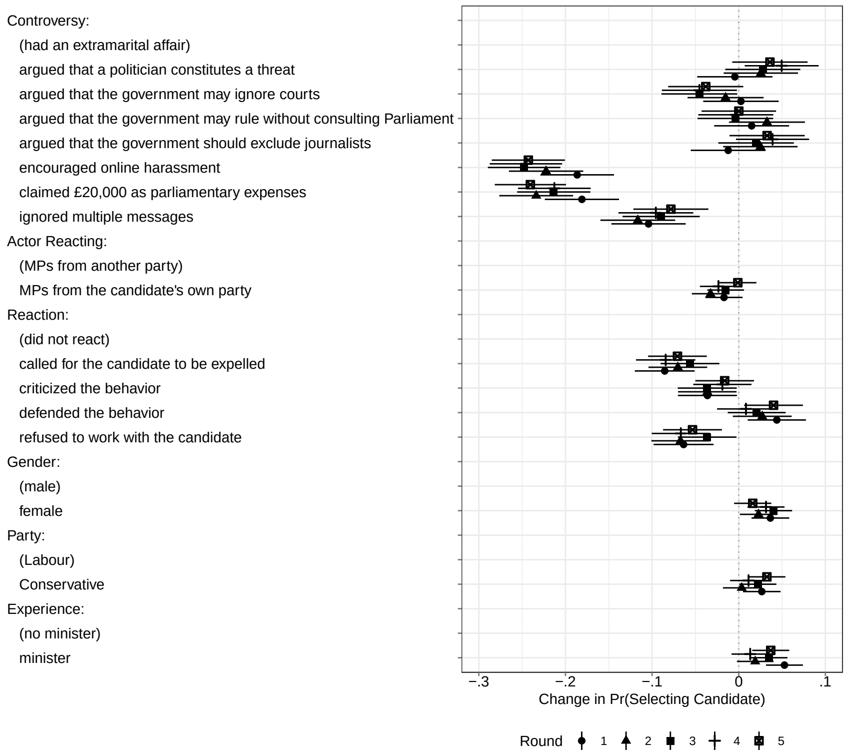

Figure A8: Conditional AMCEs by round

Similar to Bechtel and Scheve (2013), we inspect whether the randomization worked using multinomial regression models. We run one regression model for each dimension using the candidates' features as dependent variable and the respondents' features as explanatory variables. We take into account the respondents' gender, age, political views, education and income. This technique allows capturing how far the respondents' characteristics are systematically related to the candidates' features.

Tables A2 and A3 show the results of the multinomial regression models. We find that the respondents' characteristics are not systematically related to the candidates' features. More specifically, no coefficient is statistically significant at the 0.05 level. Hence, we conclude that the randomization worked successfully.

Table A2: Multinomial Regression Models I

	Gender	Party	Experience	Controversy						
	women	conservative	ministerial experience	courts	parliament	journalists	expenses	harassment	affair	messages
Age	0.001	0.001	-0.001*	0.0002	0.00002	-0.00001	0.001	0.0001	0.0001	-0.0001
	(0.001)	(0.001)	(0.001)	(0.001)	(0.001)	(0.001)	(0.001)	(0.001)	(0.001)	(0.001)
Gender	-0.022	-0.002	0.029	0.029	-0.029	0.011	-0.010	0.018	-0.013	0.011
	(0.021)	(0.021)	(0.021)	(0.041)	(0.041)	(0.041)	(0.041)	(0.041)	(0.041)	(0.041)
Conservative	-0.016	0.012	0.025	-0.016	0.035	0.002	-0.006	-0.020	0.018	0.024
	(0.021)	(0.021)	(0.021)	(0.043)	(0.043)	(0.043)	(0.043)	(0.043)	(0.043)	(0.043)
High school	-0.019	-0.013	0.012	-0.041	0.019	0.010	-0.009	-0.027	0.025	0.010
	(0.020)	(0.020)	(0.020)	(0.041)	(0.041)	(0.041)	(0.041)	(0.041)	(0.041)	(0.041)
High income	-0.019	-0.021	0.016	0.017	0.002	-0.001	-0.003	-0.015	-0.013	0.001
	(0.025)	(0.025)	(0.025)	(0.050)	(0.050)	(0.051)	(0.050)	(0.051)	(0.051)	(0.050)
Constant	0.011	-0.021	0.023	0.012	-0.005	-0.010	-0.0004	0.006	-0.015	0.008
	(0.035)	(0.035)	(0.035)	(0.070)	(0.070)	(0.070)	(0.070)	(0.070)	(0.070)	(0.070)

Note: $^{*}p<0.1$; $^{**}p<0.05$; $^{***}p<0.01$

Table A3: Multinomial Regression Models II

	Actor	Action			
	MPs same party	criticized	defended	did not react	refused
Age	-0.001	0.002*	0.002*	0.0004	0.002
	(0.001)	(0.001)	(0.001)	(0.001)	(0.001)
Gender	-0.040*	-0.005	0.016	0.056*	-0.009
	(0.021)	(0.033)	(0.032)	(0.033)	(0.033)
Conservative	-0.002	0.014	-0.023	0.017	0.018
	(0.021)	(0.034)	(0.034)	(0.034)	(0.034)
High school	-0.029	-0.041	0.026	0.012	0.005
	(0.020)	(0.032)	(0.032)	(0.032)	(0.032)
High income	0.018	0.024	0.008	-0.005	0.005
	(0.025)	(0.040)	(0.040)	(0.040)	(0.040)
Constant	0.059*	-0.072	-0.065	-0.064	-0.077
	(0.035)	(0.055)	(0.055)	(0.055)	(0.055)

Note: $^{*}p<0.1$; $^{**}p<0.05$; $^{***}p<0.01$

F Alternative Dependent Variable

The main analysis presented in the article uses the results from the question asking respondents to choose between two candidates. This section presents the results using an alternative question, which asked the respondents to rate each candidate on a seven-point scale. We estimate the AMCEs by estimating a linear regression model on the rating (Hainmueller, Hopkins and Yamamoto, 2014). Note that we rescale the rating to take values between 0 and 1.

Figure A9 illustrates the AMCEs. We find that our main findings related to hypotheses 1, 2 and 3 hold. If a candidate is countered, the probability of choosing the candidate decreases. Also, respondents provide lower ratings to candidates if the reacting actor comes from the candidate's own party. Finally, respondents give lower rating to candidate profiles with a high-cost counteraction compared to profiles with a low-cost counteraction.

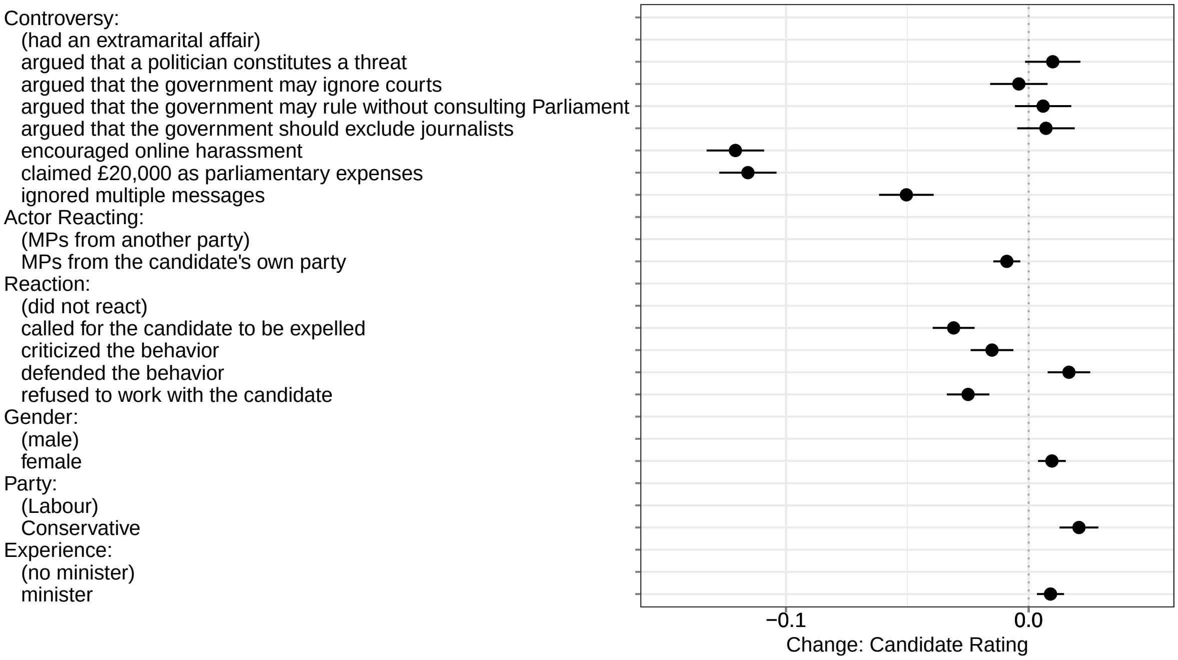

Figure A9: Effects of Candidate Features on the Candidate Rating

G Alternative Distribution of Candidate Features

In the main analysis, we assume a uniform distribution of candidate features for two reasons. First, no real-world data on the profile distributions related to controversies and counteractions is available. Our approach is thus consistent with existing studies using conjoint analysis when no real-world data is available (e.g., Bansak, Hainmueller and Hangartner, 2016). Second, the majority of our interaction effects are not statistically significant. As de la Cuesta, Egami and Imai (2022, 21) outline, the AMCE computed with a uniform distribution is externally valid if there are no interaction effects between features.

However, we conduct further analysis to assess the sensitivity of our results. We apply the model-based exploratory analysis proposed by de la Cuesta, Egami and Imai (2022), which permits estimating the population AMCEs using a different distribution of features.

Table A4 illustrates the candidates' features and the assigned probability. As the table shows, we significantly alternated the probability distribution of controversies. We assign to the controversy "Ignored multiple messages from constituents" a probability of 0.3 and to all remaining controversies a probability of 0.1. This distribution reflects the idea that ignoring multiple messages is the most frequent controversy. We assume that both features of the dimension on the reacting actor are equally likely. Furthermore, we assume that high-cost counteractions are less likely than other reactions to the controversies. Hence, we assign a probability of 0.1 to the high-cost reactions. We assign plausible probabilities to the features of gender, party, and experience as minister in light of the current composition of the House of Commons.

Table A4: Conjoint Experiment: Alternative Probability Distribution

**Dimensions**	**Features**	**Probability**
Controversy	- Argued that a politician from a different party constitutes a threat to Britain	0.1
	- Argued that the government may ignore courts in times of crisis	0.1
	- Argued that the government may rule without consulting Parliament in times of crisis	0.1
	- Argued that the government should exclude certain journalists from press briefings	0.1
	- Encouraged online harassment of a politician from a different party	0.1
	- Claimed £20,000 as parliamentary expenses for private purposes	0.1
	- Had an extramarital affair with a parliamentary assistant	0.1
	- Ignored multiple messages from constituents	0.3
Reaction: Actor	- Multiple MPs from the candidate's own party	0.5
	- Multiple MPs from another party	0.5
Reaction: Action	- Did not react to the candidate's behavior	0.2
	- Called for the candidate to be expelled from the parliamentary party on the grounds that the candidate's behavior was damaging democracy	0.1
	- Criticized the candidate's behavior for damaging democracy	0.3
	- Defended the candidate's behavior	0.3
	- Refused to work with the candidate on the grounds that the candidate's behavior was damaging to democracy	0.1
Gender	- Female	0.35
	- Male	0.65
Party	- Conservative	0.5
	- Labour	0.5
Experience as Minister	- Yes	0.3
	- No	0.7

Table A5 illustrates the population AMCEs (pAMCEs) computed using the model-based exploratory approach of de la Cuesta, Egami and Imai (2022). We focus on the

Table A5: Results of Model-based Exploratory Analysis

Dimension	Feature	pAMCE	Standard Error
Actor	MPs from the candidates own party	-0.021***	0.006
Reaction	called for the candidate to be expelled	-0.073***	0.010
Reaction	criticized the behavior	-0.041***	0.010
Reaction	defended the behavior	0.024**	0.010
Reaction	refused to work with the candidate	-0.057***	0.010

***<0.01; **<0.05; *p<0.1

attributes related to the reaction to a controversy as these are our key attributes in the paper. We find that actions from parliamentarians coming from the candidate's party and high-cost counteractions have a negative and statistically significant effect on selecting a candidate. The effect sizes of these features are also very similar to the sample AMCEs. In sum, the evidence suggests that our substantive results are robust to a different probability distribution.

H Examples of Controversies and Counteractions

In this section, we present real-world examples for the various controversies and counteractions

H.1 Controversies

Argued that a politician of a different party constitutes a threat to Britain

Example: The Conservative Party repeatedly claimed that Jeremy Corbyn, former Leader of the Labour Party, posed a serious threat to Britain's national security.

Argued that the government may ignore courts in times of crisis

Example: Multiple MPs questioned the ruling of the Supreme Court on suspending the Parliament in 2019.

Argued that the government may rule without consulting Parliament in times of crisis

Example: This controversy is related to Boris Johnson's decision to suspend Parliament during the Brexit crisis.

Argued that the government should exclude certain journalists from press briefings

Example: The UK government denied access to information to several journalists in 2020.[3]

Claimed £20,000 as parliamentary expenses for private purposes

Example: In 2009, multiple MPs from all major parties were involved in an expense scandal.

Had an extramarital affair with a parliamentary assistant

Example: Various MPs have been involved in affairs. An example is Steve Double, a Conservative politician.

Ignored multiple messages from constituents

Example: It is commonly reported that MPs do not reply to all constituent correspondence.[4]

---

[3] https://www.independent.co.uk/news/uk/politics/press-freedom-uk-government-council-of-europe-alert-boris-johnson-priti-patel-a9706741.html (last accessed: December 19, 2021).

[4] https://www.mysociety.org/2014/06/10/how-responsive-is-your-mp/ (last accessed: June 26, 2024).

H.2 Counteractions

Called for the candidate to be expelled from the parliamentary party on the grounds that the candidate's behavior was damaging to democracy

Example: Political actors in the UK frequently call for parliamentarians to be expelled from the party whip.

Criticized the candidate's behavior for damaging democracy

Example: Criticizing another MP is very common and happens regularly in PMQs.

Defended the candidate's behavior

Example: Keir Starmer backed Dawn Butler after she called Boris Johnson a liar.

Refused to work with the candidate on the grounds that the candidate's behavior was damaging to democracy

Example: Lord Young quit government after Boris Johnson prorogued Parliament in 2019.

I Questionnaire

I.1 Pre-Treatment

Party Identification

Question: *Generally speaking, do you think of yourself as Labour, Conservative, Liberal Democrat or what?*

Answer:

- Conservative

- Labour
- Liberal Democrat
- Other Party
- None

Question (if the answer is "None"): *Do you generally think of yourself as a little closer to one of the parties than the others?Which one?*

Answer:

- Conservative
- Labour
- Liberal Democrat
- Other Party

Authoritarian personality: Childrearing

Question: *Let's now move to another topic. Although there are a number of qualities that people feel children should have, every person thinks that some are more important than others. We will show you some desirable qualities. Please rate which of these qualities is most important to you using the 1 to 5 scale below.*

Answer:

- A child is independent (1).....A child has respect for the elderly (5)
- A child is curious (1).... A child is good mannered (5)
- A child is obedient (1) ....A child is self-reliant (5)
- A child is considerate (1)....A child is well behaved (5)

Pluralism

Question: *Please rate how much you agree with the following statements.*

- *In a democracy it is important to make compromises among differing viewpoints*
- *It is important to listen to the opinion of other groups.*
- *Diversity limits my freedom.*

Answer:

- 1 (I very much disagree) - 5 (I very much agree)

I.2 Survey Experiment

Experimental Treatment

We present respondents with the following introductory text:

> *Now imagine an election where you face a choice between two political candidates standing for re-election to Parliament. We would like your opinion on these candidates.*
>
> *You will see information about a series of candidates. The table below gives you information about the first two candidates.*
>
> *Politicians are sometimes involved in minor or major controversies, and these candidates have been both been criticised for some aspect of their behaviour or views in the past. We also give you details about other aspects of their experiences and background.*
>
> *Please review the information in the table very carefully.*

We then show the respondents information about two candidates, varying the information along six dimensions (see Table 1 in the manuscript). We focus on respondents in England. In Table A6, we illustrate an example of the conjoint experiment:

Table A6: Conjoint Experiment: Example

	**Candidate A**	**Candidate B**
Gender	Female	Male
Party	Conservative	Labour
Experience as Minister	Yes	No
Controversy	Had an extramarital affair with a parliamentary assistant	Argued that the government should exclude certain journalists from press briefings
Reaction	Multiple MPs from another party criticised the candidate's behaviour for damaging democracy	Multiple MPs from another party called for the candidate to be expelled from the parliamentary party on the grounds that the candidate's behaviour was damaging to democracy

After the respondents have been presented with the two candidates, they were asked which of the two they prefer, and also asked to rank both candidates on a 7-point scale. This will be repeated 5 times in total.

Question: *If you had to say, which of the two candidates do you prefer?*

Answer:

- Candidate A
- Candidate B

Question: *On a scale from 1 to 7, where 1 indicates that you would never support this*

candidate, and 7 indicates that you would always support this candidate, where would you place each of the candidates?

Answer:

- 1 (Never support) - 7 (Always support)

I.3 Post-Treatment

Question: *How important is it for you, if at all, to live in a country that is governed democratically? On this scale where 1 means it is 'not at all important' and 10 means 'absolutely important' what position would you choose?*

Answer:

- 1 (Not at all important) - 10 (Absolutely important)

Question: *Politicians are sometimes involved in controversies. Below we have listed some examples from around the world. To what degree do you think each of these behaviors are a threat to democracy, if at all? Use this scale where 1 means 'no threat at at all to democracy' and 7 means 'a serious threat to democracy'.*

- A politician argued that the government may ignore courts in times of crisis
- A politician argued that the government may rule without consulting Parliament in times of crisis.
- A politician argued that a politician of a different party constitutes a threat to Britain.
- A politician encouraged online harassment of a politician from a different party.
- A politician argued that the government should exclude certain journalists from press briefings.

- A politician claimed £20,000 as parliamentary expenses for private purposes
- A politician had an extramarital affair with a parliamentary assistant
- A politician ignored multiple messages from constituents

Answer:

- 1 (no threat at all to democracy) - 10 (a serious threat to democracy)

References

Akkerman, Agnes, Cas Mudde and Andrej Zaslove. 2014. "How Populist Are the People? Measuring Populist Attitudes in Voters." *Comparative Political Studies* 47(9):1324–1353.

Bansak, Kirk, Jens Hainmueller and Dominik Hangartner. 2016. "How Economic, Humanitarian, and Religious Concerns Shape European Attitudes toward Asylum Seekers." *Science* 354(6309):217–222.

Bechtel, Michael and Michael Scheve. 2013. "Mass Support for Global Climate Agreements Depends on Institutional Design." *Proceedings of the National Academy of Sciences* 110(34):13763–13768.

de la Cuesta, Brandon, Naoki Egami and Kosuke Imai. 2022. "Improving the External Validity of Conjoint Analysis: The Essential Role of Profile Distribubtion." *Political Analysis* 30(1):19–45.

Egami, Naoki and Kosuke Imai. 2019. "Causal Interaction in Factorial Experiments: Application to Conjoint Analysis." *Journal of the American Statistical Association* 114(526):529–540.

Feldman, Stanley and Karen Stenner. 1997. "Perceived Threat and Authoritarianism." *Political Psychology* 18(4):741–770.

Frederiksen, Kristian Vrede Skaaning. 2022. "Does Competence Make Citizens Tolerate Undemocratic Behavior?" *American Political Science Review* 116(3):1147–1153.

Graham, Matthew and Milan W. Svolik. 2020. "Democracy in America? Partisanship, Polarization, and the Robustness of Support for Democracy in the United States." *American Political Science Review* 114(2):392–409.

Hainmueller, Jens, Daniel Hopkins and Teppei Yamamoto. 2014. "Causal Inference in Conjoint Analysis: Understanding Multidimensional Choices via Stated Preference Experiments." *Political Analysis* 22(1):1–30.

Krishnarajan, Suthan. 2023. "Rationalizing Democracy: The Perceptual Bias and (Un)Democratic Behavior." *American Political Science Review* 117(2):474–496.

Leeper, Thomas, Sara Hobolt and James Tilley. 2020. "Measuring Subgroup Preferences in Conjoint Experiments." *Political Analysis* 28(2):207–221.

Stenner, Karen. 2005. *The Authoritarian Dynamic*. Cambridge: Cambridge University Press.
